# Neutrophil-derived Il1r2 modulates inflammation and alleviates acute lung injury by promoting M2 macrophage polarization

**DOI:** 10.1038/s41598-025-14278-4

**Published:** 2025-11-10

**Authors:** Weiwei Ding, Hui Zhang, Bing Li, Xiaodong Xu, Yitian Yang, Luyao Zhang

**Affiliations:** Department of Anesthesiology and Perioperative Medicine, Henan Provincial People’s Hospital, People’s Hospital of Zhengzhou University, Zhengzhou, Henan 450003 China

**Keywords:** Acute lung injury, Bioinformatics analysis, Machine learning, Immunity, Il1r2, Acute inflammation, Chemokines

## Abstract

Acute Lung Injury (ALI) and Acute Respiratory Distress Syndrome (ARDS) are severe inflammatory conditions with high morbidity and mortality. Understanding the molecular mechanisms underlying these diseases is crucial for developing effective treatments. To investigate the molecular mechanisms underlying ALI, we established a lipopolysaccharide (LPS)-induced mouse model. Bioinformatics and machine learning techniques were utilized to identify key genes and construct gene co-expression networks. Single-cell RNA sequencing was performed to analyze Il1r2 expression specifically in neutrophils. CellChat and hdWGCNA were employed to explore gene co-expression modules and cell-cell communication networks, respectively. Experimental validations included qRT-PCR for gene expression quantification, and western blotting, immunohistochemistry, and immunofluorescence for protein-level confirmation. Four key genes—Cebpd, Hspa12b, Pim1, and Il1r2—were identified as potential biomarkers and therapeutic targets. Il1r2 was identified as a key regulator of inflammation, predominantly expressed in neutrophils. Immune cell infiltration analysis revealed increased neutrophils, monocytes, and dendritic cells in ALI samples. CellChat and hdWGCNA highlighted the significant role of Il1r2 in neutrophil-macrophage signaling in immune regulation. Furthermore, overexpression of Il1r2 in neutrophils reduced lung inflammation and promoted M2 macrophage polarization in vivo. This indicated that Il1r2 alleviates ALI by modulating the immune response, particularly through interactions with macrophages. Neutrophil-derived Il1r2 plays a critical role in modulating inflammation in ALI by promoting M2 macrophage polarization. These findings suggest that targeting Il1r2 may offer a novel therapeutic approach to control the immune response in ALI and other inflammatory diseases.

**Supplementary Information:**

The online version contains supplementary material available at 10.1038/s41598-025-14278-4.

## Introduction

Acute Lung Injury (ALI) and Acute Respiratory Distress Syndrome (ARDS) are severe pulmonary conditions characterized by acute onset, hypoxemia, and bilateral infiltrates visible on chest imaging, presenting significant clinical challenges due to their high morbidity and mortality rates^1,2^. Both conditions can arise from a variety of direct and indirect insults to the lungs, leading to widespread inflammation and increased vascular permeability^3,4^. This results in non-cardiogenic pulmonary edema and impaired gas exchange. Common causes include infections^5,6^ (bacterial, viral, and fungal), such as pneumonia, sepsis, influenza, and COVID-19^7–9^; exposure to lipopolysaccharides (LPS); inflammation from acute pancreatitis; and major surgeries, particularly cardiac or organ transplantation^10,11^. Current treatments for ALI/ARDS include mechanical ventilation, fluid management, prone positioning, corticosteroids, and supportive care^12^. While these approaches can provide critical support and improve outcomes, no cure currently exists. Therefore, developing new anti-inflammatory drugs, identifying biomarkers for early diagnosis and prognosis, and personalizing treatment strategies are crucial. These efforts aim to enhance treatment effectiveness and improve patient outcomes.

In recent years, advances in bioinformatics and machine learning have enabled more efficient and unbiased identification of key molecular players in complex diseases like ALI^13,14^. Machine learning can manage vast and complex datasets, identifying patterns and predicting outcomes that might be overlooked by traditional methods^15^. This capability facilitates efficient analysis of diverse biological data, leading to more accurate and comprehensive insights. Single-cell analysis offers a high-resolution view of cellular heterogeneity, revealing the distinct roles and responses of individual cells within the lung^16^. Combining these techniques enables researchers to uncover intricate cellular mechanisms and interactions, thereby deepening the understanding of ALI pathophysiology and potentially guiding the development of targeted therapies. Liu et al. explored the role of oxidative stress-related genes in ALI and conducted immune infiltration-related analyses using bioinformatics tools^17^. Wang et al. systematically revealed the cellular transcriptional landscape of lung tissue in sepsis-induced lung injury, underscoring the power of these advanced techniques^18^. Additionally, a series of studies have investigated the mechanisms and treatments of ALI using multiple machine learning models and single-cell resolution^19,20^.

Leveraging these computational tools, we identified interleukin-1 receptor type 2 (Il1r2) as a potential regulator involved in ALI pathogenesis. Il1r2 is a decoy receptor that binds interleukin-1 (IL-1) cytokines without transducing intracellular signals, acting as a negative regulator of IL-1-mediated inflammation^21^. By sequestering IL-1α and IL-1β, Il1r2 limits excessive inflammatory responses and protects tissues from damage caused by uncontrolled cytokine activity. Previous studies have demonstrated that Il1r2 expression is upregulated in various inflammatory conditions and serves as a key modulator in diseases such as rheumatoid arthritis^22^, sepsis^23^, and heart injury^24^. However, the precise molecular mechanisms and cell-type-specific functions of Il1r2 in ALI/ARDS remain incompletely understood, highlighting the need for further investigation.

In this study, we utilized well-established bioinformatics tools and transcriptional profiling data from ALI mouse samples and normal control mouse lung tissues obtained from the GEO database to identify potential biomarkers for ALI/ARDS. We also examined the enrichment pathways and immune infiltration mechanisms related to these genes. Finally, we identified potential diagnostic markers for ALI/ARDS and compared their immunological features and molecular mechanisms. This comprehensive approach provides deeper insights into the molecular pathophysiology of ALI/ARDS and establishes a theoretical foundation for early detection and targeted treatment.

## Materials and methods

### Data collection

The datasets were obtained from the GEO database (https://www.ncbi.nlm.nih.gov/geo/). The microarray datasets included samples from both ALI and healthy states, with detailed information provided in Table S1. The GSE6730, GSE2411, GSE269740, and GSE222957 datasets, used as the analysis set, were background corrected, quantile normalized, and merged into a combined dataset. Batch effects were then removed using the SVA package (version 3.42.0)^25^. The GSE235367 and GSE216943 datasets were used as the validation set.

### Identification of differentially expressed genes (DEGs)

DEGs between the pediatric sepsis group and the control group were identified using the limma package^26^. To reduce false positives caused by multiple testing, adjusted p-values (false discovery rate, FDR) were calculated using the GEO platform. Genes were considered significantly differentially expressed if they met the following cutoff criteria: adjusted *P* < 0.05 and absolute log2 fold change (|log2FC|) > 1^27^. A volcano plot was generated to visualize these DEGs. Subsequently, the top 50 DEGs based on statistical significance and fold change were selected for heatmap visualization using the pheatmap package in R.

### Functional enrichment analysis

To investigate the functions and pathways of the overlapping DEGs, we conducted functional enrichment analysis using the R software packages clusterProfiler V3.18.1^28^ and Goplot V1.0.2, with significance thresholds of *P* < 0.05 and q-value < 0.05. Gene Ontology (GO) terms, including biological process (BP), cellular component (CC), and molecular function (MF), as well as Kyoto Encyclopedia of Genes and Genomes (KEGG) pathways, were analyzed and visualized. Further insights into the biological significance of DEGs were obtained using the Metascape database (http://metascape.org), while pathway enrichment analysis utilized the Molecular Signatures Database (MSigDB) Hallmark Gene Sets and KEGG Pathways^29^. Gene Set Enrichment Analysis (GSEA) was conducted with the GSEA software, applying a significance criterion of *P* < 0.05^30^. Additionally, Gene Set Variation Analysis (GSVA) was performed (version 1.32.0) on our dataset to identify enriched cellular pathways^31^.

### Weighted gene Co-Expression network analysis (WGCNA)

The WGCNA approach was employed to construct and modularize gene networks using the R package WGCNA (version 1.7.3)^32^. The combined dataset, including both ALI and control samples, was used as trait data to identify key genes. Initially, samples were clustered, and outlier samples were removed to ensure accuracy. A sample cluster and heatmap of clinical traits were then constructed. The soft threshold of the data was determined to ensure the gene interactions conformed to a scale-free distribution. Phylogenetic clustering trees among genes were based on adjacency relationships and gene similarity. The minimum number of genes per module was set to 200 using the hybrid dynamic tree cutting algorithm. Modules with the highest disease relevance and the key genes they contained were selected. Estimates of module membership (MM) and gene significance (GS) were used to connect modules with clinical features. Modules showing the highest Pearson correlation with MM and statistical significance (*P* < 0.05) were defined as hub modules. Criteria of MM > 0.8 and GS > 0.2 were used to identify highly connected and clinically relevant modules. Gene information for these associated modules was provided for further research.

### High dimentional weighted gene co-expression network analysis (hdWGCNA)

hdWGCNA to identify key genes associated with monocytes and macrophages in lung injury samples. Monocyte and macrophage clusters were selected from scRNA data, followed by the construction of a gene expression correlation matrix, weighted gene co-expression network, and module detection. Module-trait relationship analysis identified modules significantly correlated with the ALI group, and central genes within these modules were determined based on connectivity. In ALI samples, the top 25 central genes were identified as key genes associated with neutrophil-related macrophages (M6).

### Screening and validation of characteristic markers

Novel and significant biomarkers for ALI were identified using three machine learning algorithms: random forests^33^ (RF), least absolute shrinkage and selection operator^34^ (LASSO) logistic regression, and support vector machine-recursive feature elimination^35^ (SVM-RFE). The “randomForest” R package was used for RF analysis, while LASSO logistic regression was performed with the R package “glmnet” with the optimal model determined by the minimal lambda. Genes identified as overlapping across RF, LASSO, and SVM-RFE were selected as candidate biological markers. To assess the diagnostic value of these biomarkers for ALI, ROC curves and the area under the curve (AUC) were calculated using the R software package pROC (V1.18.0)^36^. Statistical significance was set at a two-sided *P* < 0.05. Subsequently, the expression levels of these biomarkers were validated in the GSE216943 dataset to evaluate their potential as diagnostic biomarkers.

### Assessment and correlation of immune cell infiltration profiles

Using the Cell-type identification by estimating relative subsets of RNA transcripts (CIBERSORT) tool^37^, immune cell infiltration matrices for 22 different immune cell types were obtained, with a significance threshold of *P* < 0.05. CIBERSORT, a deconvolution algorithm, analyzed the gene expression data, applying linear support vector regression to deconvolve the bulk expression matrix and reduce noise. This method enabled precise quantification of immune cell infiltration in each ALI gene expression profile. Only samples with a CIBERSORT output of *P* < 0.05 were considered for further analysis. Correlations between candidate diagnostic biomarkers and significantly altered immune cells were assessed using Spearman correlation coefficients, with the results visualized using the ‘reshape2’ and ‘ggExtra’ packages in R.

### Processing and analysis of single-cell transcriptome data

Raw data for GSE235367 and GSE20751 were downloaded from the GEO database. Single-cell transcriptome data were first preprocessed using Cellranger (10X Genomics)^38^. The data were then imported into R and analyzed with the Seurat V4.1.0 package. Quality control was performed by filtering cells based on the criteria: gene count per cell > 200 and < 2500, and mitochondrial gene percentage < 20%. The data were normalized using the NormalizeData function, followed by the selection of the top 2000 variably expressed genes using the “vst” method in the FindVariableFeatures function. Data were scaled with the ScaleData function before performing principal component analysis (PCA), clustering, and Uniform Manifold Approximation and Projection (UMAP) dimensionality reduction using the RunPCA, FindClusters, and RunUMAP functions. Cell clusters were visualized with UMAP plots created by the DimPlot function, and differential expression of markers was determined with the FindAllMarkers function, with violin plots generated by the VlnPlot function. Cell type annotation was performed using the SingleR V1.4.1 package with the celldex V1.0.0 reference database. To ensure accurate annotation, identified clusters were validated by examining the expression of canonical marker genes known from the literature^39–41^. This validation step confirmed the reliability of the cell type assignments.

To infer the differentiation status within neutrophil subpopulations, we applied the CytoTRACE V0.3.3 R package^42^. Based on gene expression counts, CytoTRACE calculated stemness scores for each cell, with higher scores indicating lower differentiation. These scores were visualized on UMAP plots and used to objectively define the root state in the trajectory analysis of neutrophil subsets. Subclustering analysis of the neutrophil cluster, pseudo-temporal trajectory analysis using “Monocle2” and cell-cell communication analysis using “CellChat” were performed.

### LPS-induced ALI model in mice

To develop an LPS-induced ALI model, C57BL/6 mice were randomly divided into two groups. One group was intraperitoneally injected with a single dose of PBS mixed with LPS (10 mg/kg; Sigma Aldrich, Cedex, France), while the control group received PBS alone. Outcome measures included the lung injury index, with the primary outcome being the expression levels of core genes. The experimental unit was a single mouse. Sample size was determined based on prior studies and power calculations to ensure statistical significance^43^. Inclusion criteria required mice to be within a specific age and weight range, and free from underlying health conditions; exclusion criteria were established a priori to exclude any mice displaying abnormal baseline characteristics. Randomisation was used to allocate mice to groups using a computer-generated sequence, and blinding was maintained throughout allocation, experiment conduct, outcome assessment, and data analysis. After 20 h, mice were anesthetized with an intraperitoneal injection of 1% pentobarbital (50 mg/kg), followed by euthanasia through cervical dislocation. Lung tissues were then harvested for quantitative real-time PCR analysis^43^. All procedures involving animal experimentation were conducted following the ethical guidelines approved by the Animal Care and Use Committee of Zhengzhou University (ZZULAC2021092804), adhering to ARRIVE guidelines for in vivo research. Animal euthanasia was performed in accordance with the American Veterinary Medical Association (AVMA) Guidelines for the Euthanasia of Animals (2020).

### Il1r2 overexpression in the lungs

To induce Il1r2 overexpression in mouse lungs, a recombinant AAV9-Il1r2 vector (mm-Il1r2-GCAGAGAGUUCAAAUCUGAdTdT) and a control vector AAV9-GFP were purchased from Shanghai HanHeng Co. (Shanghai, China). The viral titers for both constructs were approximately 1 × 10¹² encapsidated genomes/mL. Male C57BL/6J mice (5–7 weeks old) were anesthetized with 0.3% pentobarbital sodium (25 mL/kg). Each mouse received an intravenous tail-vein injection of 40 µL of either AAV9-GFP or AAV9-Il1r2. Mice were then allowed to recover and were maintained under standard housing conditions.

After three weeks, lung tissues were harvested for subsequent analysis. Four distinct groups were established for comparison: Con + AAV9-GFP; Con + AAV9-Il1r2; ALI + AAV9-GFP; ALI + AAV9-Il1r2. Il1r2 expression in lung tissues was assessed by western blotting and immunofluorescence staining to confirm successful overexpression.

### Quantitative real time polymerase chain reaction (qRT-PCR)

Total RNA was extracted with Trizol reagent (15596026, Invitrogen) and detected by real-time PCR. RNA was reverse transcribed into cDNA using PrimeScript RT kit (A15300, Invitrogen). qPCR experiments were performed using a SYBR Green PCR kit (4367659, Applied biosystems). β-actin was used as an internal control^44^. The primer sequences of target genes including Cebpd, Hspa12b, Pim1, Il1r2 and β-actin are shown in Table S2.

### Western blotting

Homogenized mouse lung tissues were lysed with radioimmunoprecipitation assay (RIPA) lysis buffer (Solarbio, R0020) containing a protease inhibitor cocktail (Beyotime, P1030). Protein concentration was determined using the Pierce BCA Protein Assay Kit (Thermo Fisher, USA). Cell lysates were boiled and then separated by SDS-PAGE, followed by transfer onto polyvinylidene difluoride (PVDF) membranes (Bio-Rad, USA). The membranes were blocked with 5% nonfat dry milk in tris-buffered saline with 0.1% Tween 20 for 1 h at room temperature, and incubated with primary antibodies overnight at 4 °C. The following primary antibodies were used: iNOS (1:1000, Abcam), CD86 (1:1000, Abcam), GAPDH (1:5000, CST), CD206 (1:1000, Abcam), Arg-1 (1:1000, Abcam), TNF-α (1:1000, CST), IL-1β (1:1000, CST), Il1r2 (1:1000, Novus Biologicals), and Pim2 (1:1000, Abcam). After washing three times with TBST (2% Tween), the membranes were incubated with HRP-conjugated secondary antibodies for 1 h at room temperature. Protein bands were detected using an enhanced chemiluminescence kit (Thermo Fisher, USA) and quantified using ImageJ software (NIH, USA).

### Immunohistochemistry

For histological analysis, bronchoalveolar lavage fluid (BALF) was not collected. Instead, the left lung lobe was excised, fixed in 10% neutral-buffered formalin, and embedded in paraffin for tissue sectioning. The paraffin-embedded tissue was cut into 5-µm sections, which were then flattened onto slides, dehydrated, and mounted. Additionally, lungs were fixed via formalin perfusion, embedded in paraffin, and sectioned into 5-µm slices. Sections were incubated overnight with primary anti-Il1r2 antibodies, followed by treatment with a secondary antibody. Immunohistochemistry images were captured using an optical microscope (BX51; Olympus, Tokyo, Japan)^12^.

For quantification, three random high-power fields (HPFs) per section were selected from each mouse (*n* = 3 per group). Il1r2-positive cells were manually counted in each field using ImageJ software based on DAB (brown) staining. The average number of positive cells per field was calculated for each mouse. Results are presented as mean ± standard deviation (SD), and statistical significance between groups was assessed using one-way ANOVA with post hoc testing.

### Immunofluorescence staining

Lung tissue sections were deparaffinized in xylene and rehydrated through a graded ethanol series, then rinsed in distilled water and PBS. Antigen retrieval was performed by heating the slides in citric acid buffer (pH 6.0). After cooling, sections were blocked with 5% normal goat serum or bovine serum albumin in PBS for 1 h. Primary antibody incubation was carried out overnight at 4 °C with anti-Ly6G (1:200) to identify neutrophils and anti-Il1r2 (1:200) for Il1r2 detection. Following primary antibody incubation, sections were washed and then incubated with fluorescently labeled secondary antibodies for 1 h in the dark. Nuclear staining was performed with DAPI for 5 min. Slides were mounted with fluorescence mounting medium, and fluorescence images were captured using a fluorescence microscope (BX51; Olympus, Tokyo, Japan) with appropriate filter settings. The images were analyzed to evaluate the localization and expression of Ly6G (ab307167; abcam) and Il1r2 (ab212208; abcam)^12^.

For quantification, co-localized Il1r2⁺Ly6G⁺double-positive cells were manually counted using ImageJ software in three randomly selected high-power fields per section for each mouse (*n* = 3 per group). The average number of double-positive cells per field was calculated per mouse. Data are presented as mean ± SD, and statistical comparisons between groups were performed using one-way ANOVA.

### Statistical analysis

All statistical analyses were performed using GraphPad Prism (version 8.0.1, GraphPad Software, Inc., San Diego, CA), R software (version 3.6.0), and SPSS^14^. For experimental comparisons, two-tailed unpaired Student’s *t*-tests were used for two groups, while one-way or two-way ANOVA followed by Bonferroni’s post hoc test was applied for multiple group comparisons. Data normality was assessed using the Shapiro–Wilk test. All quantitative data are presented as mean ± SD, and *P* < 0.05 was considered statistically significant. Unless otherwise stated, experiments were conducted independently in triplicate. For bioinformatics analyses involving multiple hypothesis testing — such as the identification of DEGs, GO enrichment, and WGCNA — false discovery rate (FDR) correction was applied using the Benjamini – Hochberg method. Adjusted *P*-values (FDR < 0.05) were considered statistically significant and are reported throughout the main text, figure legends, and supplementary tables.

## Result

### DEGs identification and functional enrichment analysis

**Fig. 1:**
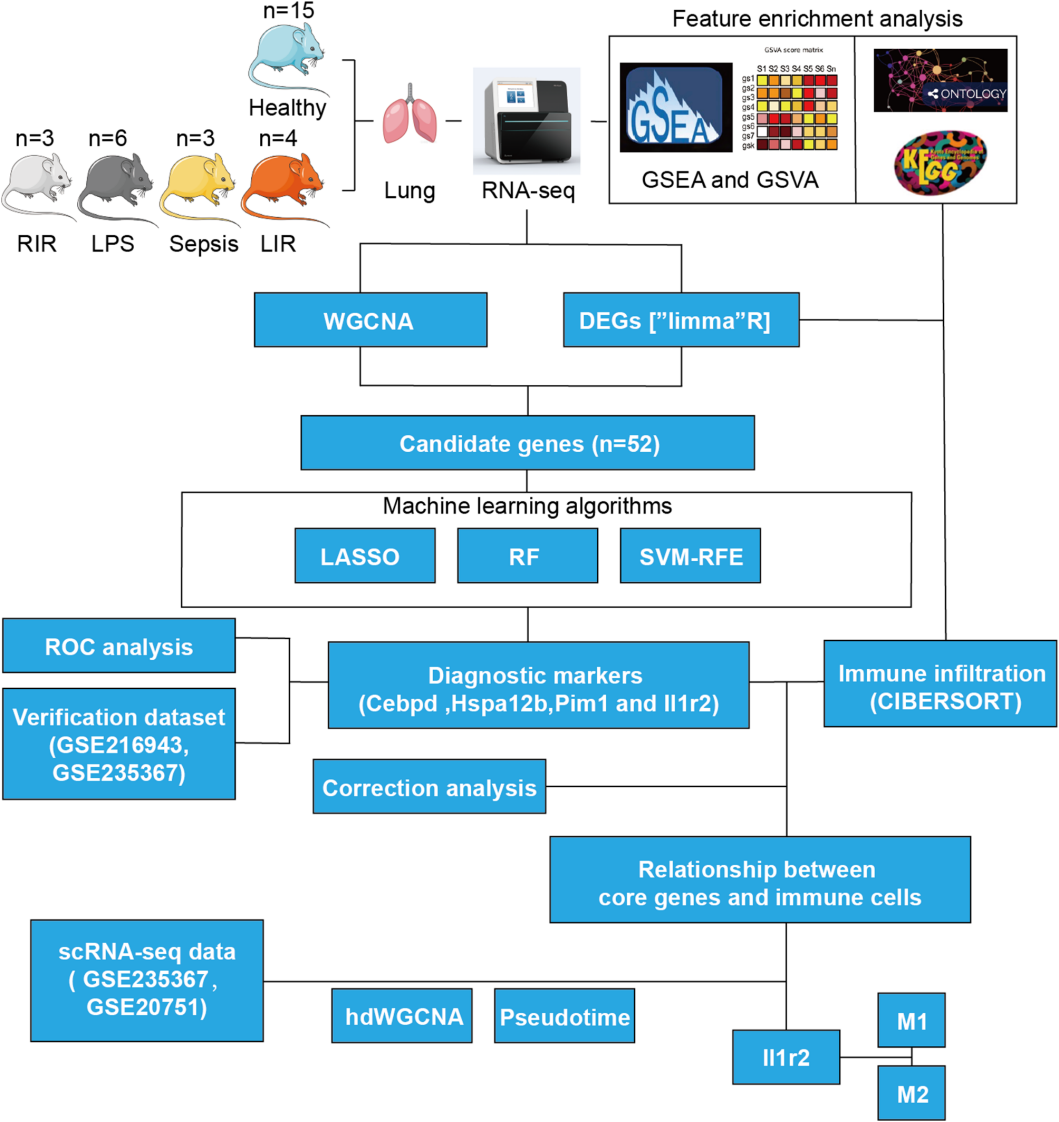
Flowchart illustrating the gene set analysis process. RIR, Renal ischemia reperfusion. LPS, Lipopolysaccharide. LIR, lung ischemia reperfusion. DEGs, Differentially Expressed Genes. GSEA, Gene Set Enrichment Analysis. GSVA, Gene Set Variation Analysis. GO, Gene Ontology. KEGG, Kyoto Encyclopedia of Genes and Genomes. LASSO, Least Absolute Shrinkage and Selection Operator. RF, Random Forest. SVM-RFE, Support Vector Machine-Recursive Feature Elimination. ROC, Receiver Operating Characteristic Curve. CIBERSORT, Cell-type Identification By Estimating Relative Subsets Of RNA Transcripts. WGCNA, Weighted Gene Co-Expression Network Analysis. scRNA-seq, Single-cell RNA sequencing.

The primary study design is illustrated in Fig. 1. Differential expression analysis revealed that in GSE6730, a total of 402 DEGs were identified, including 220 upregulated and 182 downregulated genes. Similarly, in GSE2411, 250 DEGs were identified, with 234 upregulated and 16 downregulated. The analysis of GSE269740 identified 2,540 DEGs, with 1,071 upregulated and 1,469 downregulated genes. Likewise, GSE222956 contained 277 DEGs, with 192 upregulated and 85 downregulated. The distribution of these DEGs is shown in the volcano plots (Figure S1A). Subsequently, the top 50 genes from each of the four datasets were displayed in heatmaps (Figure S1B).

Further functional enrichment analysis revealed significant upregulation of the “myeloid leukocyte migration” pathway across all four datasets in the ALI group. Additionally, the “granulocyte migration” pathway was notably upregulated in the ALI group in GSE2411, GSE269740, and GSE222956 (Figure S2A-D). The top five GO-enriched pathways and their associated genes for each dataset were also identified and are presented in Figure S2E-F.

### Screening for DEGs between ALI and control samples

**Fig. 2:**
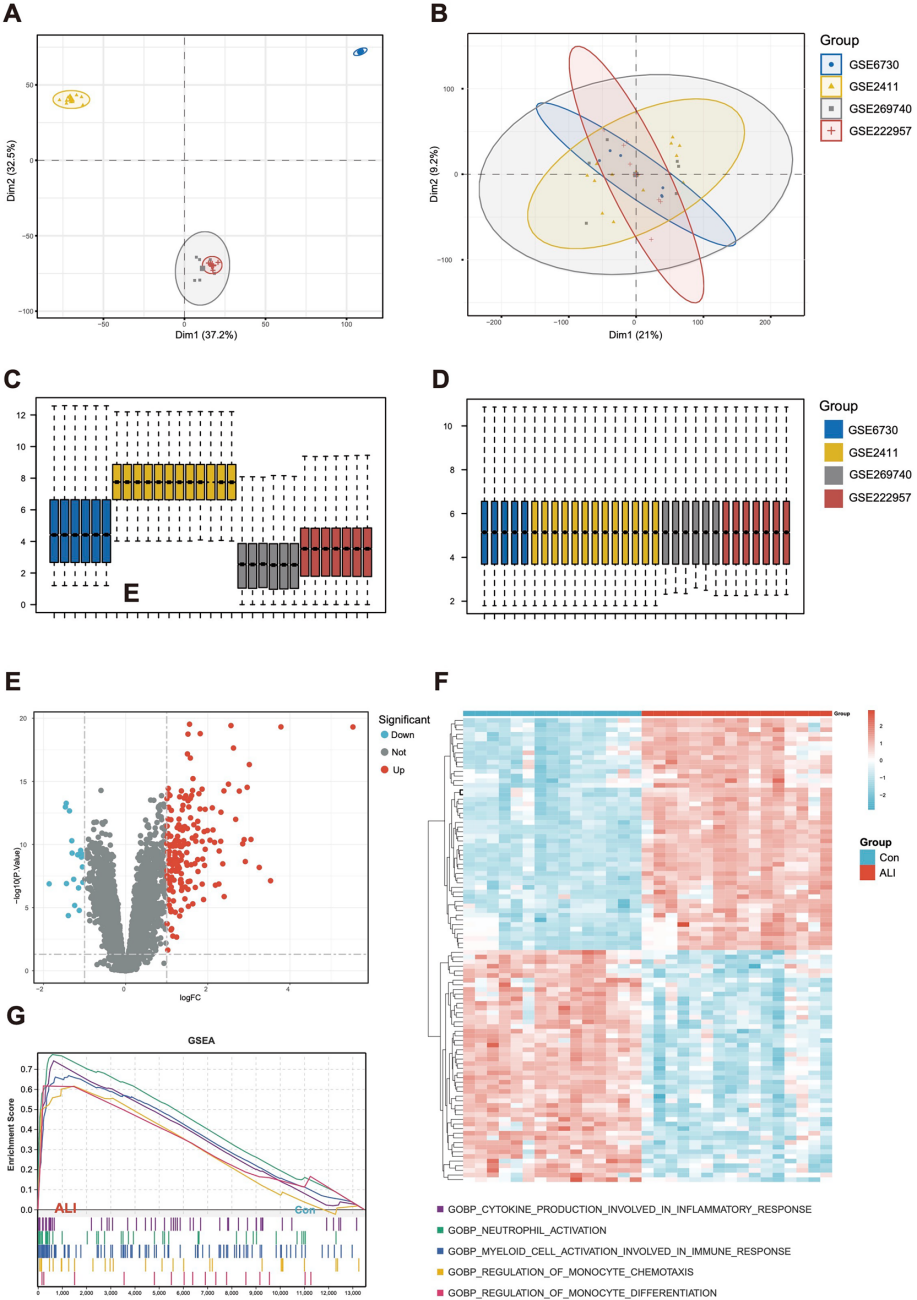
Identification of DEGs in lung tissue. (**A**) PCA for ALI and Con samples before batch correction with ComBat. (**B**) PCA for ALI and Con samples after batch correction with ComBat. (**C**) Box line diagram of the merged dataset before correction. (**D**) Box line diagram of the combined dataset after correction. (**E**) Volcano plot showing DEGs between ALI and Con samples. (**F**) Heatmap showing the top 50 up- and down-regulated genes (**G**) Active GO functional enrichment of gene expression matrix in ALI. The differentially expressed genes were selected with log2 (fold change) > 1 or log2 (fold change) < − 1 and with statistical significance (*P* < 0.05). Two-tailed unpaired Student’s t-test for two groups or one-way ANOVA for three groups or more. DEGs, Differentially Expressed Genes. PCA, Principal Component Analysis. GO, Gene Ontology. ALI, Acute Lung Injury. Con, Control.

Batch correction was applied to the consolidated datasets from the four GEO series to mitigate batch effects (Fig. 2A-D). The DEGs between the ALI and control groups are visually represented through a heatmap and a volcano plot (Fig. 2E-F). In addition, the GSEA results demonstrated that in the lung tissue gene expression matrix, active GO functions are mainly enriched in biological processes including activation of production involved in inflammatory response, neutrophil activation, and myeloid cell activation involved in immune response and regulation of monocyte chemotaxis (Fig. 2G, Figure S3).

### Weighted gene co-expression network construction

**Fig. 3:**
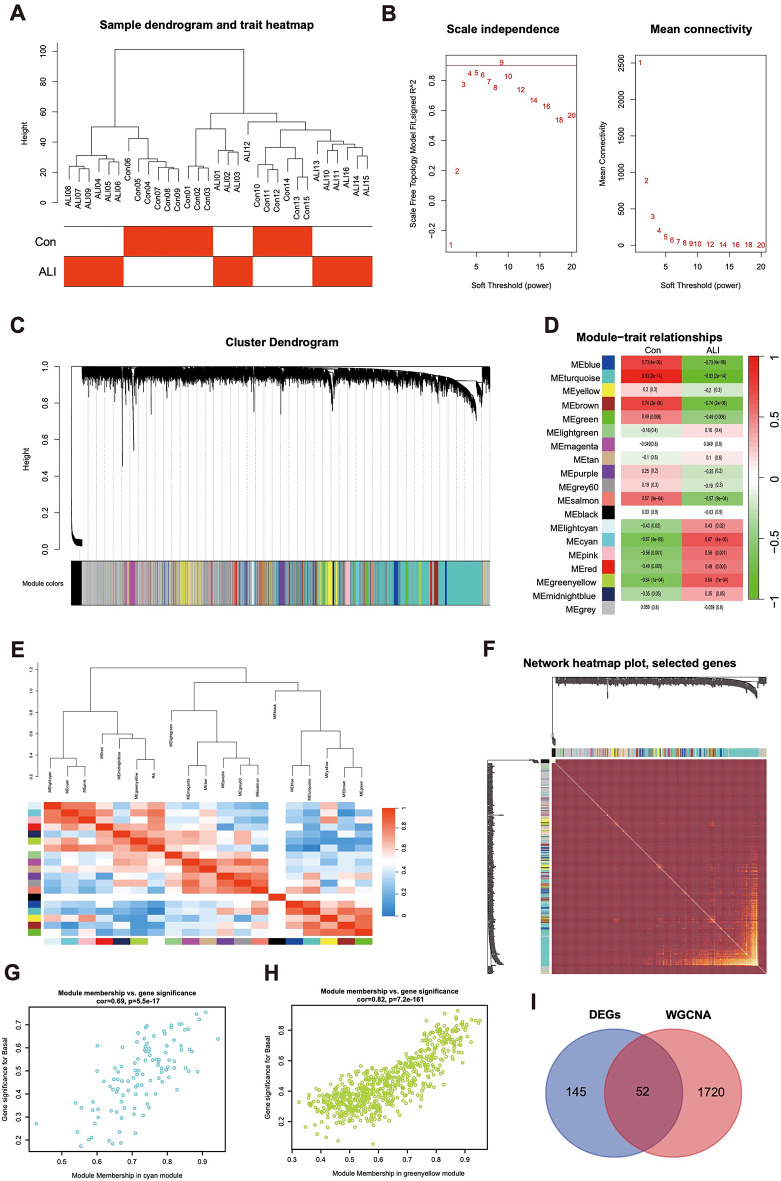
Construction of WGCNA co-expression network. (**A**) Sample clustering dendrogram with tree leaves corresponding to individual samples. (**B**) The screen of the best soft thresholds. Nine was considered the best soft threshold. (**C**) The merging of similar modules. (**D**) Correlations between different modules and clinical traits. Red represents a positive correlation, and blue represents a negative correlation. (**E**) Collinear heat map of module feature genes. Red color indicates a high correlation, blue color indicates opposite results. (**F**) Clustering dendrogram of module feature genes. (**G**,**H**) The significance of genes related to ALI in the brown and cyan module (a dot represents the genes in the module). (**I**) A Venn diagram illustrated the overlap between diagnostic markers identified through DEGs and WGCNA. DEGs, Differentially Expressed Genes. ALI, Acute Lung Injury. WGCNA, Weighted Gene Co-Expression Network Analysis.

We performed a WGCNA on the multi-chip dataset, selecting 15 control samples and 16 ALI samples to cluster the samples and remove obvious outliers by setting a threshold, as shown in Fig. 3A. Next, as depicted in Fig. 3B, we set the soft threshold to 9, based on an *R*^*2*^ value greater than 0.90 and high average connectivity. After merging strongly correlated modules using a 0.25 clustering height cutoff, 19 modules were selected for further analysis, as displayed in the clustering dendrogram (Fig. 3C). The correlations between module eigengenes (MEs) and clinical traits were examined to evaluate the association of gene modules with ALI-related features (Fig. 3D, Table S3,4). A transcriptional correlation analysis within modules confirmed the reliability of module delineation, showing no significant inter-module correlations (Fig. 3E, F). Further analysis indicated that the turquoise, blue, brown, green, and salmon modules were negatively correlated with ALI, while the cyan, greenyellow, pink, red, lightcyan, and midnightblue modules exhibited positive associations with ALI (Fig. 3G-H, Figure S4A). A more detailed investigation of all genes within the identified modules revealed 52 overlapping genes between the major module genes from WGCNA and DEGs, as shown in the Venn diagram (Fig. 3I, Figure S4B).

### Identification of characteristic genes via machine learning algorithms

**Fig. 4:**
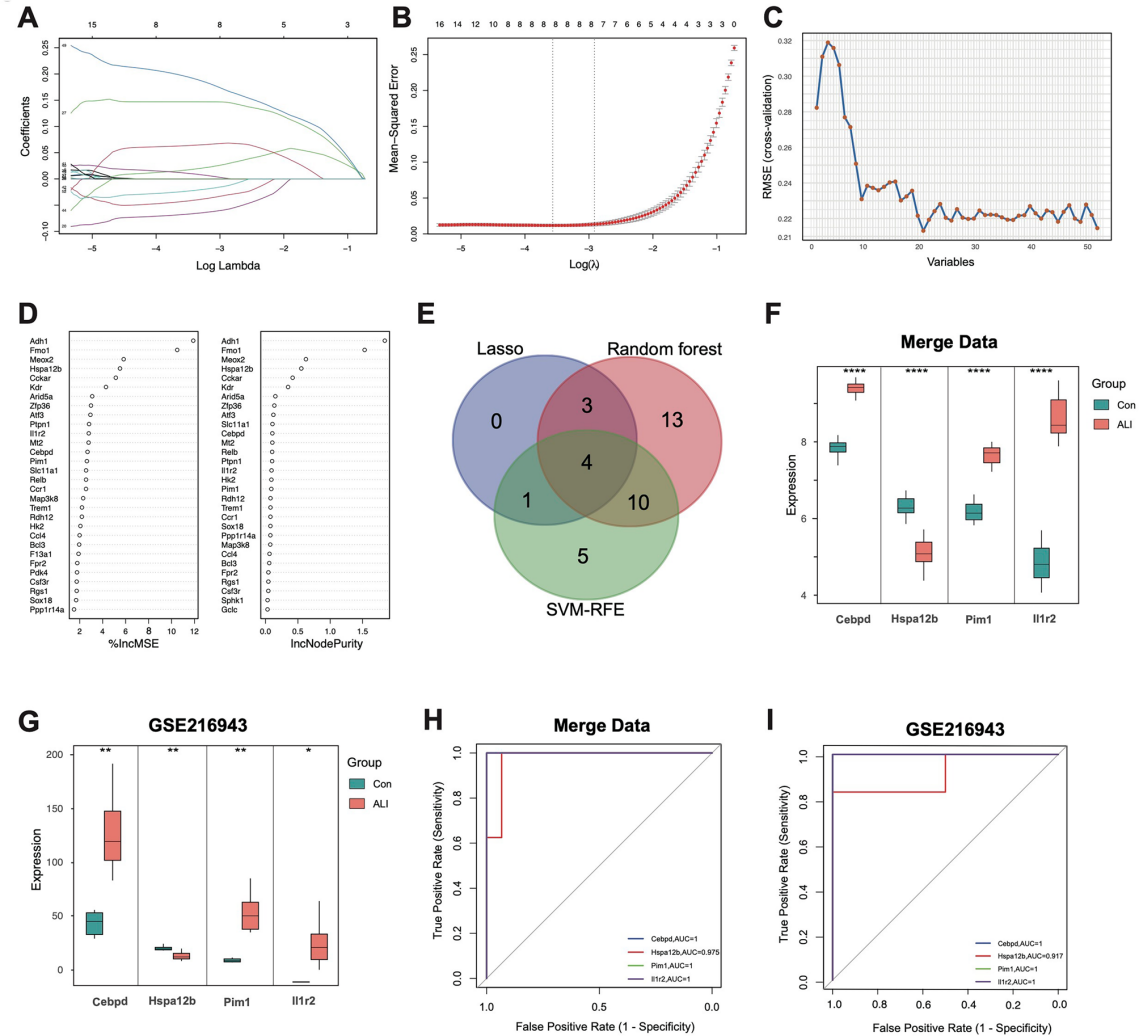
The selection of characteristic genes of ALI via machine learning algorithm. (**A**, **B**) LASSO analysis of the combined dataset. (**C**) Biomarkers were screened based on SVM-RFE. (**D**) Based on RF algorithm to screen biomarkers. (**E**) A Venn diagram illustrated the overlap between diagnostic markers identified through machine learning algorithm. (**F**) Boxplot showed the expression of hub genes between ALI and control group in combined dataset. (**G**) Boxplot showed the expression of hub genes between ALI and control group in GSE216943. (**H**) The ROC curve of the diagnostic efficacy verification. (**I**) The ROC curve of the diagnostic efficacy verification in GSE216943. *P*-values were calculated as mean ± SD, *P* < 0.05 were considered statistically significant differences. *P < 0.05; **P < 0.01; ***P < 0.005. Two-tailed unpaired Student’s t-test for two groups or one-way ANOVA for three groups or more. ALI, Acute Lung Injury. LASSO, Least Absolute Shrinkage and Selection Operator. RF, Random Forest. SVM-RFE, Support Vector Machine-Recursive Feature Elimination. ROC, Receiver Operating Characteristic Curve.

Candidate diagnostic biomarkers were identified using three different machine learning algorithms. The LASSO logistic regression algorithm was applied to select 9 meaningful feature variables associated with ALI from the DEGs (Fig. 4A-B). RF with feature selection was then used to determine the relationship between error rate and the number of classification trees, selecting the top 20 genes based on their weight (Fig. 4C). SVM-RFE algorithm was employed to classify the 52 features among all DEGs, identifying a subset of 20 significant features (Fig. 4D). A Venn diagram analysis was performed to pinpoint common genes across the three algorithms, leading to the identification of four genes: Cebpd, Hspa12b, Pim1, and Il1r2 (Fig. 4E).

Next, we analyzed the expression levels of these four genes in the merged dataset, which showed significantly different expression between the control and ALI groups (Fig. 4F). The GSE216943 dataset was then used as an external validation cohort to verify both the analytic results and the expression levels of the four candidate biomarkers (Fig. 4G). In this dataset, Cebpd, Hspa12b, Pim1, and Il1r2 also showed significantly different expression between the control and ALI samples.

To further assess the diagnostic efficacy of these four genes, we validated them using ROC analysis on both the merged dataset and the GSE216943 dataset. ROC analysis, the gold standard for evaluating diagnostic accuracy and survival rates, confirmed the diagnostic value of these genes, with the Area Under the Curve (AUC) for all four genes in both datasets showing strong diagnostic potential (Fig. 4H-I).

### Analysis of immune cell infltration

**Fig. 5:**
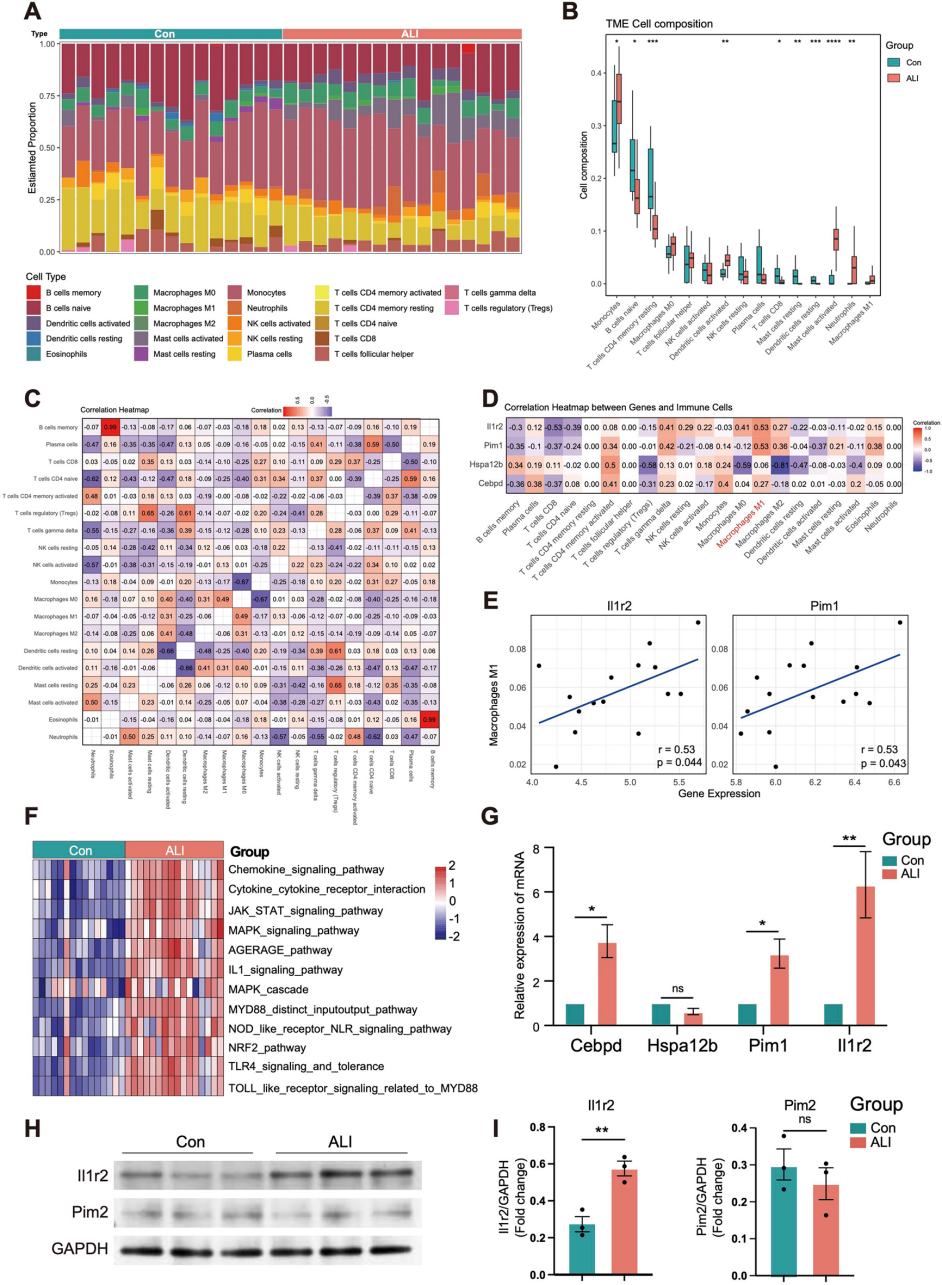
Immune cell infiltration analysis in the combined dataset. (A) Distribution of 22 kinds of immune cells in tissues of ALI and control groups. (B) Expression of immune cells in ALI and control groups. (C) Correlation diagram between immune cells. (D) Correlations between Cebpd, Hspa12b, Pim1, Il1r2 and infltrating immune cells. (E) Correlations between Pim1,Il1r2 and Macrophages M1. (F) GSVA enrichment analysis results in ALI and control groups. (G) The boxplot shows the expression of hub genes (Cebpd, Hspa12b, Pim1 and Il1r2) between the ALI and control groups as measured by qRT-PCR. (H) Immunoblotting to assess the expression levels of Pim1 and Il1r2 in lung tissue from control (*n* = 3) and ALI (*n* = 3). (I) The boxplot shows the expression levels of Pim1 and Il1r2 in lung tissue from control (*n* = 3) and ALI (*n* = 3). Two-tailed unpaired Student’s t-test for two groups or one-way ANOVA for three groups or more. GSVA, Gene Set Variation Analysis. ALI, Acute Lung Injury.

Using the CIBERSORT algorithm, we first calculated the proportion of immune cell infiltration between the control and ALI groups (Fig. 5A). The results showed that the infiltration of monocytes, activated dendritic cells, activated mast cells, and neutrophils were significantly increased in ALI samples compared to control samples. In contrast, control samples showed a higher proportion of infiltration by naive B cells, resting memory CD4^+^T cells, and CD8^+^T cells (Fig. 5B).

Further analysis revealed the correlations between the 19 types of infiltrating immune cells. Activated neutrophils were positively correlated with activated mast cells and activated memory CD4^+^T cells but negatively correlated with naive CD4^+^T cells and activated NK cells. Additionally, activated dendritic cells showed significant positive correlations with both M1 and M2 macrophages (Fig. 5C).

To explore the relationships between key genes and infiltrating immune cells in lung injury, we performed a correlation analysis. Both Il1r2 and Pim1 showed strong positive correlations with M1 and M2 macrophages and negative correlations with CD8^+^T cells (Fig. 5D-E).

To further understand the underlying biological mechanisms, we performed GSEA using 21,338 gene sets from the MSigDB resource. The results indicated that myeloid cell activation in immune response and neutrophil activation may play significant roles in the development of ALI (Figure S5). Additionally, GSVA revealed that the chemokine signaling pathway, Toll-like receptor signaling via MYD88, JAK-STAT signaling pathway, MAPK signaling pathway, and IL-1 signaling pathway were primarily enriched in ALI samples (Fig. 5F). Next, we performed qRT-PCR to analyze the expression patterns of four key genes in the ALI group. The results showed that the levels of Cebpd, Pim1, and Il1r2 were significantly elevated in ALI samples, while the expression of Hspa12b was significantly decreased under LPS treatment (Fig. 5G). Additionally, Western blot analysis revealed a significant increase in Il1r2 levels in the ALI group (Fig. 5H, I, Figure S6A-C).

### Expression levels of key genes in single cell transcriptome data

**Fig. 6:**
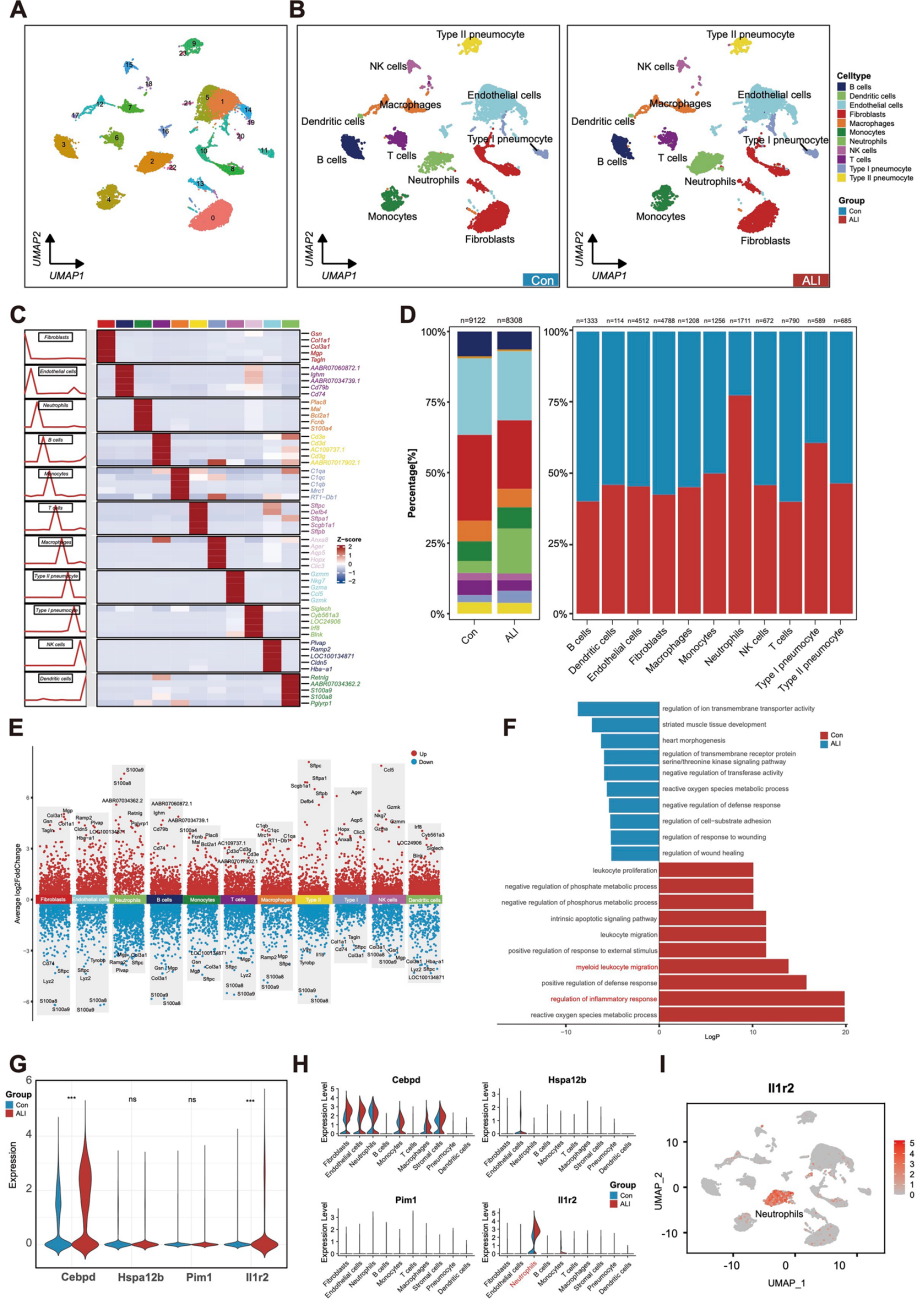
Key genes expression analysis in the single-cell transcriptome dataset GSE235367. (**A**,**B**) UMAP visualization of all cells in 23 clusters. Each dot represents a cell whose color is coded according to the different clusters. Clusters are named by the most specific and highly cross-gene interpretation. (**C**) The top5 expressed marker genes for each cell type. (**D**) Histogram indicated the proportion of cells in the lung tissue for both analyzed samples. (**E**) The scatter plot shows the top 5 and bottom 5 expression marker genes for each cell type. (**F**) GO enrichment analysis results in ALI and control samples. (**G**) Violin plot illustrates the expression levels of four core genes (Cebpd, Hspa12b, Pim1 and Il1r2) in ALI and control samples. (**H**) Violin plot illustrates the expression levels of four core genes (Cebpd, Hspa12b, Pim1 and Il1r2) in each cell type. (**I**) The UMAP plots illustrates the expression distribution of Il1r2. p-values were calculated as mean ± SD, *P* < 0.05 were considered statistically significant differences. *P < 0.05; **P < 0.01; ***P < 0.005. Two-tailed unpaired Student’s *t* - test for two groups or one-way ANOVA for three groups or more. GO, Gene Ontology. ALI, Acute Lung Injury. UMAP, Uniform Manifold Approximation and Projection.

The datasets GSE235367 and GSE20751 were downloaded from the NCBI GEO database, and several preprocessing steps were applied, including normalization, scaling, clustering, and screening for highly variable genes. The dimensionality-reduced clusters were visualized on a 2D map generated using UMAP after PCA based on 2000 highly variable genes (Fig. 6A-B). Using the expression profiles of classic marker genes and the SingleR database, we merged clusters with similar gene expression patterns and identified 11 major cell types from the integrated data: fibroblasts, endothelial cells (ECs), monocytes, macrophages, dendritic cells, neutrophils, B cells, NK cells, T cells, Type I pneumocytes, and Type II pneumocytes (Fig. 6C).

The cell proportions in samples from both groups revealed that fibroblasts and ECs were the most prominent subpopulations. Additionally, the proportions of neutrophils and Type I pneumocytes were higher in the ALI group compared to controls (Fig. 6D). Figure 6E shows the distribution of the top 5 DEGs for each cell type. GO enrichment analysis revealed that pathways related to the “regulation of inflammatory response” and “myeloid leukocyte migration” were significantly upregulated in the ALI samples (Fig. 6F). The expression differences of key genes between the two groups are shown in Fig. 6G, highlighting that Cebpd and Il1r2 were significantly upregulated in the ALI group (*P* < 0.05). To further explore the distribution of core genes, violin plots were generated (Fig. 6H). Notably, Il1r2 expression was significantly elevated in neutrophils following ALI (Fig. 6I).

### Identification and characterization of neutrophil subpopulations

**Fig. 7:**
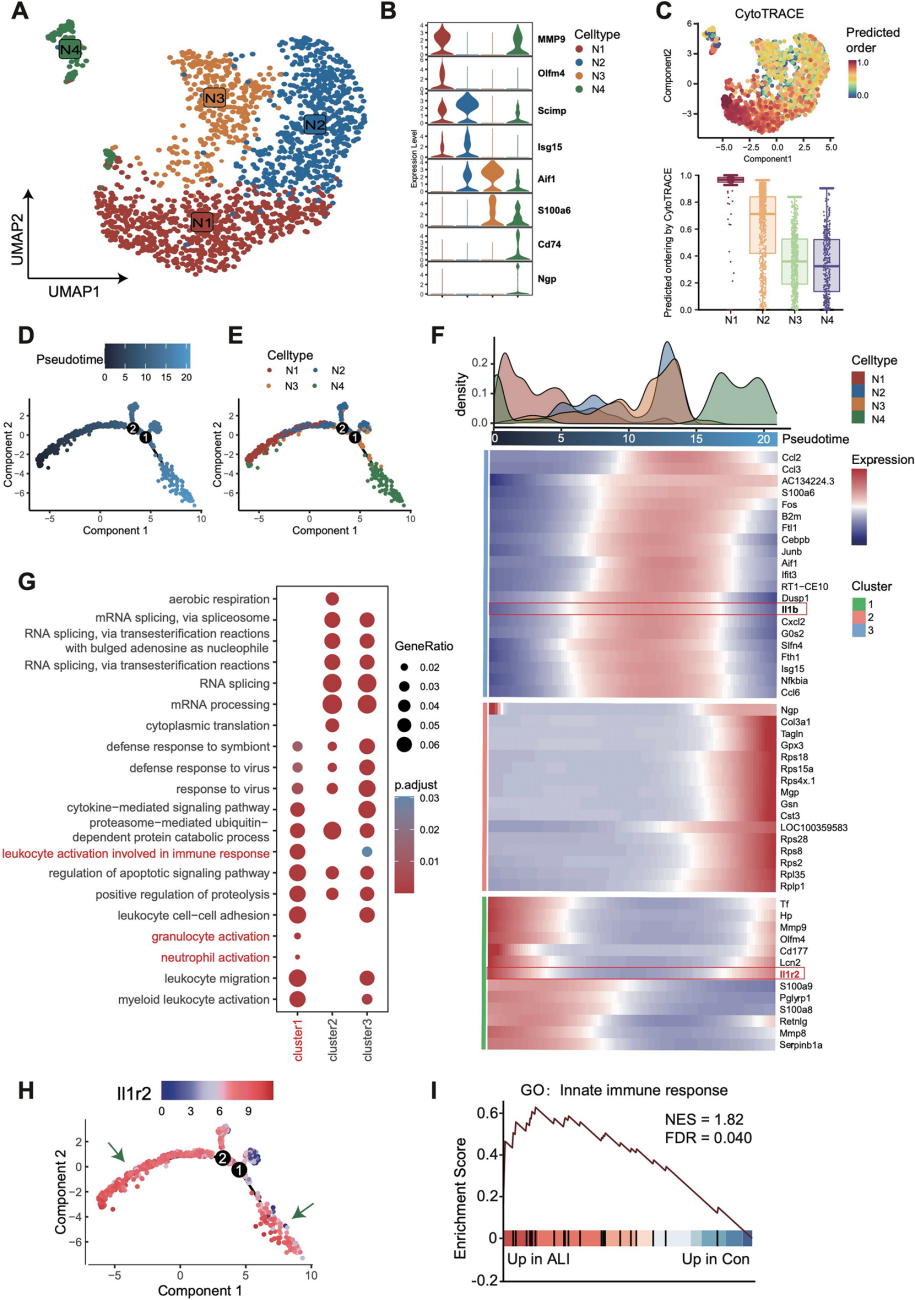
Comparison of neutrophil subpopulations in the lung tissue of mice. (**A**) UMAP profiles of neutrophil subpopulations. (**B**) Violin plot illustrates the expression levels of top2 genes in each cluster. (**C**) UMAP plot (top) and boxplot (bottom) showing CytoTRACE scores. Higher scores reflect lower differentiation status. (**D**,**E**) The trajectory of differentiation of neutrophils subgroup. Dots: individual cells, color: cell type or temporal state. (**F**) Heatmap about the dynamics of gene expression during differentiation. The distribution of neutrophil subtypes during the transition can be divided into 3 stages and pseudo-times. (**G**) GO pathway enrichment analysis of DEGs among neutrophil subtypes. (**H**) The expression levels of Il1r2 in different differentiation stages. (**I**) GSEA enrichment analysis results in ALI samples. ALI, Acute Lung Injury. UMAP, Uniform Manifold Approximation and Projection. DEGs, Differentially Expressed Genes. GO, Gene Ontology. GSEA, Gene Set Enrichment Analysis.

The neutrophils were reclustered and annotated into 4 distinct celltypes (Fig. 7A). Canonical lineage markers were employed for unbiased clustering Fig. 7B). To further validate the choice of the root cluster for trajectory analysis, we applied CytoTRACE, a computational method that predicts developmental potential based on gene expression profiles. CytoTRACE analysis identified the N1 cluster as having the highest developmental potential, confirming it as the progenitor population and supporting its selection as the root for trajectory inference (Fig. 7C; Figure S5A). Consistent with this, the N4 cluster was enriched at the opposite terminal of the inferred developmental trajectory, representing a more differentiated state (Fig. 7D, E; Figure S5B). Expression of canonical inflammation signatures was detected in all 3 subtypes (Fig. 7F). To further explore the functional characteristics of each differentiation stage, we conducted GO pathway enrichment analysis. This revealed that neutrophils were activated at the C1 stage, while immune response-related cytokines were activated in both the C1 and C3 stages (Fig. 7G). Notably, Il1r2 was highly expressed in the both the C1 and C3 stages (Fig. 7H). GSEA further demonstrated significant upregulation of the “innate immune response” in the ALI group (Fig. 7I).

### High-dimensional weighted gene co-expression network analysis

**Fig. 8:**
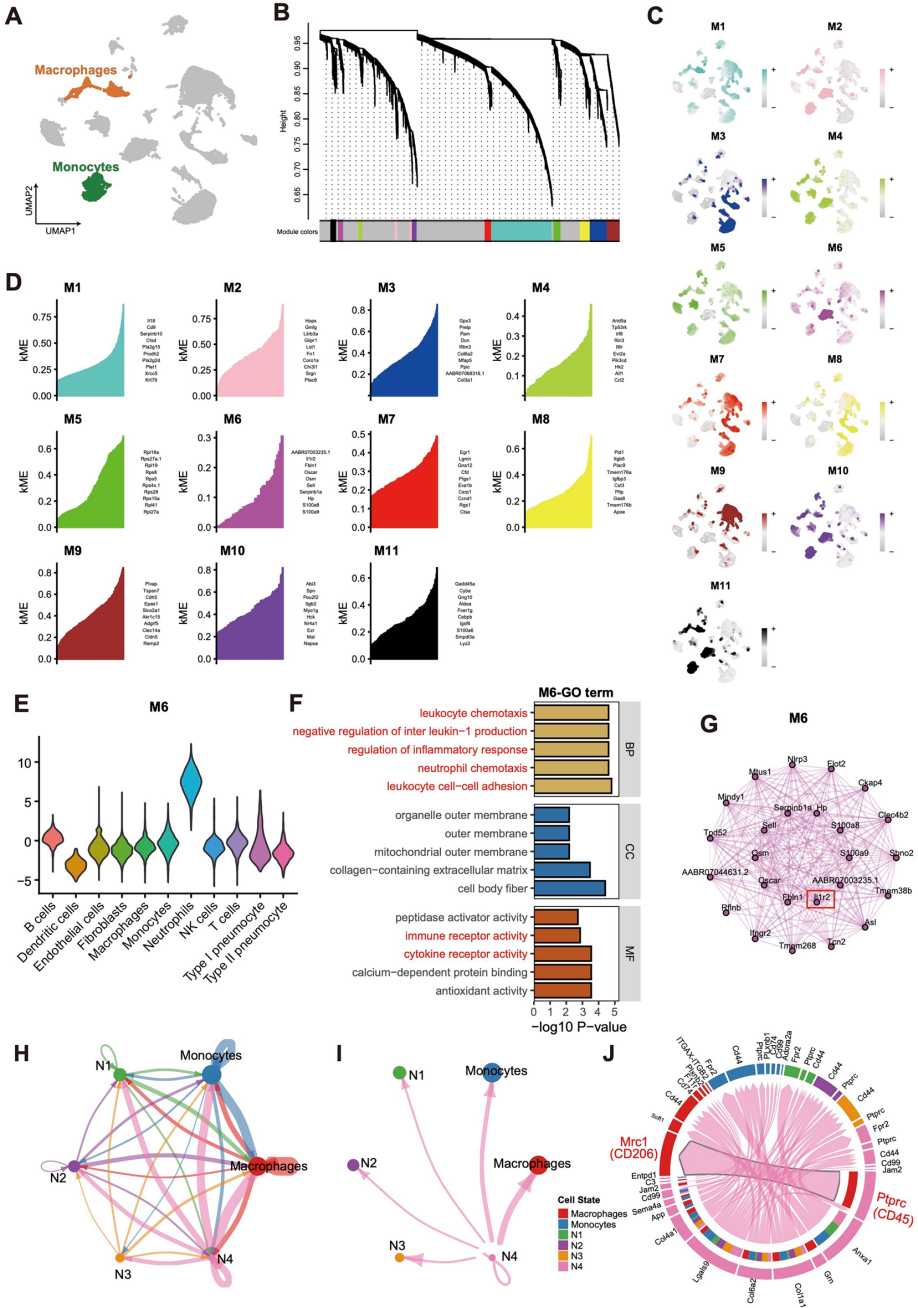
The WGCNA analysis of macrophage cells. (**A**) UMAP plot of monocytes and macrophages. (**B**) Construction of co-expression network using the optimal soft threshold of 6, with genes divided into 11 modules and resulting in a dendrogram. (**C**) UMAP plot of macrophages with ME staining. (**D**) KMes for each module characterisation gene. (**E**) Violin plot showing the gene set scores of the M6 in various celltypes. (**F**) Barplot showing the most significantly enriched GO pathway in M6. (**G**) Gene network of M6. (**H**) Communication interactions network plot for neutrophils, monocytes, and macrophages. (**I**) Communication interaction network plot for N4 neutrophils and other cell types. (**J**) Chord diagram for neutrophils, monocytes, and macrophages. The length of the arc represents the interaction count. A larger size means more interaction with other cell types. WGCNA, Weighted Gene Co-Expression Network Analysis. UMAP, Uniform Manifold Approximation and Projection. GO, Gene Ontology.

We used hdWGCNA to identify the key molecular features of primary macrophages(Fig. 8A). During the construction of the co-expression network, we observed that when the scale-free topology fitness index reached 0.90, the soft threshold power β was 6 (Figure S5C) to build an unweighted primary macrophages cell network, achieving the best connectivity. We identified 11 gene modules (Fig. 8B). The correlation between the 11 modules is shown in Figure S5D. And we calculated modular connectivity to determine the connectivity of each gene based on the characterised genes (Fig. 8C, D). Subsequently, we assessed the module scores and found that the M6 was highly activated in neutrophils (Fig. 8E). Next, we selected key genes from the M6 subtype for enrichment analysis (Fig. 8F) and extracted the top 25 genes for PPI network analysis (Fig. 8G). To distinguish the cells of interest, we integrated the monocyte/macrophage and neutrophil subpopulations. Subsequently, cell-cell communication analysis was performed using the ‘CellChat’ package. The communication results revealed that the N4 subgroup and macrophages were significantly involved in the Ptprc–Mrc1 signaling pathway (Fig. 8H-I).

### Overexpression of Il1r2 alleviates M1 polarization and reduces lung inflammation

**Fig. 9:**
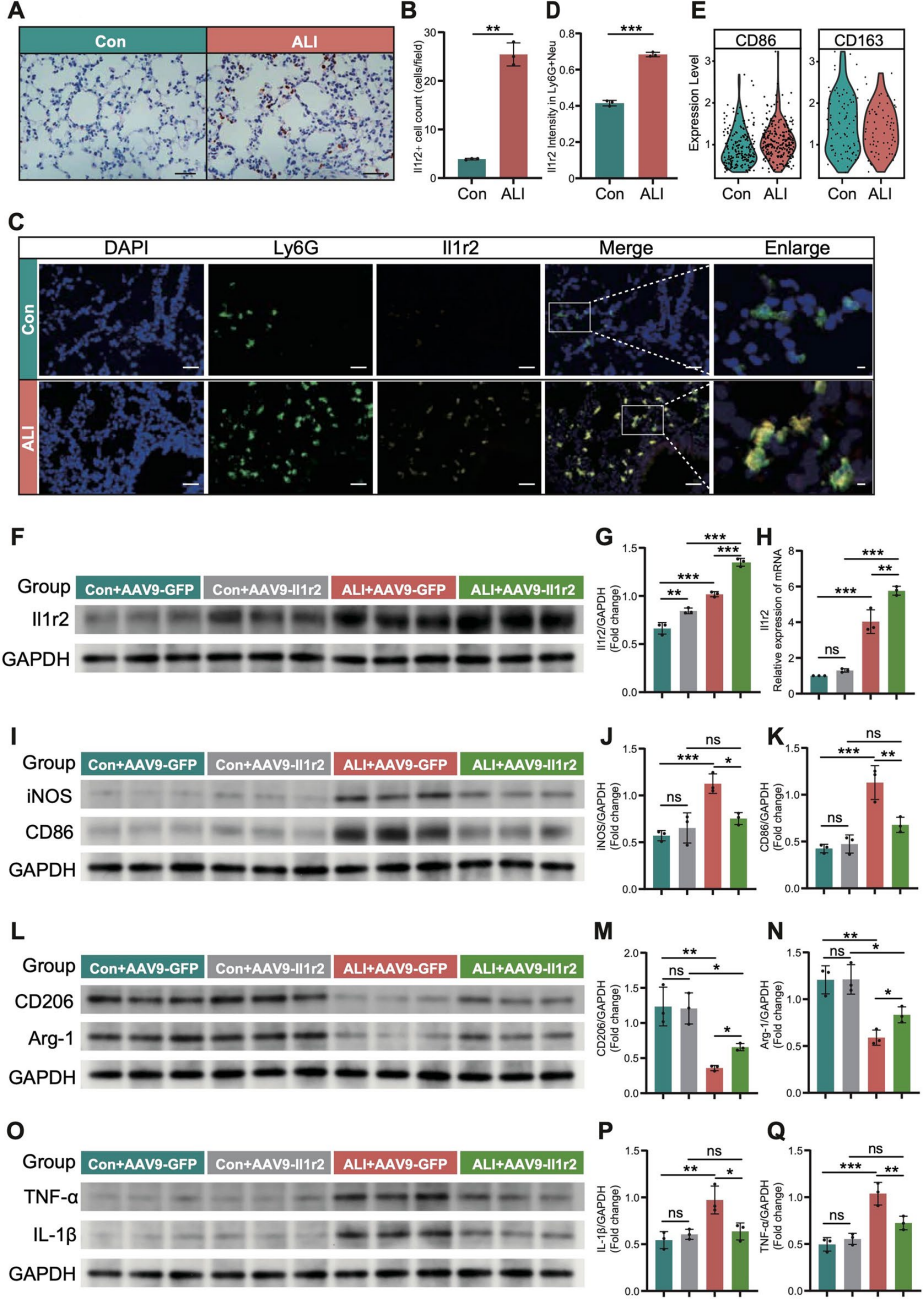
Neutrophil-derived Il1r2 promotes M2 polarization in ALI. (**A**) Representative immunohistological staining images demonstrating Il1r2 expression in the lungs of mice. Scale bar, 100 μm. (**B**) Quantification of Il1r2 expression levels based on immunohistochemistry (*n* = 3 mice per group) (**C**) Immunofluorescence staining was used to verify Il1r2 secretion by neutrophils in ALI samples. Scale bars: 100 μm and 20 μm. (**D**) Quantification of Il1r2 expression based on immunofluorescence (*n* = 3 mice per group). (**E**) Violin plot showing the expression levels of Cd86 and Cd163 in ALI and control samples. (**F**) Immunoblotting analysis of Il1r2 expression in lung tissues from Con, Con + Il1r2-OE, ALI, and ALI + Il1r2-OE mice (*n* = 3 mice per group). (**G**) Densitometric quantification of Il1r2 protein levels in (**F**). (**H**) RT-qPCR analysis of Il1r2 mRNA expression in lung tissues from Con + AAV9-GFP, Con + AAV9-Il1r2, ALI + AAV9-GFP, and ALI + AAV9-Il1r2 mice (*n* = 3 mice per group). (**I**–**K**) Immunoblotting and quantification of iNOS and CD68 protein levels in lung tissues from Con + AAV9-GFP, Con + AAV9-Il1r2, ALI + AAV9-GFP, and ALI + AAV9-Il1r2 mice (*n* = 3 mice per group). (**L**–**N**) Immunoblotting and quantification of CD206 and Arg-1 expression in lung tissues from Con + AAV9-GFP, Con + AAV9-Il1r2, ALI + AAV9-GFP, and ALI + AAV9-Il1r2 mice (*n* = 3 mice per group). (**O**–**Q**) Immunoblotting and quantification of TNF-α and IL-1β expression in lung tissues from Con + AAV9-GFP, Con + AAV9-Il1r2, ALI + AAV9-GFP, and ALI + AAV9-Il1r2 mice (*n* = 3 mice per group). All data are expressed as mean ± SD, *P* < 0.05 were considered statistically significant differences. *P < 0.05; **P < 0.01; ***P < 0.005. Two-tailed unpaired Student’s t-test for two groups or one-way ANOVA for three groups or more. Con, Control; ALI, Acute Lung Injury.

Immunohistochemistry confirmed the elevated expression of Il1r2 in the ALI group (Fig. 9A, B). Given the crucial role of neutrophils in ALI models^2^, particularly in the early inflammatory response, immunofluorescence co-localization techniques were utilized to examine Il1r2 expression in these cells (Fig. 9C, D). Findings indicated that, concurrent with lung injury, neutrophils not only became activated but also secreted higher levels of Il1r2. The communication results indicated a significant upregulation of the Ptprc-Mrc1 pathway between the N4 subgroup and macrophages (Figure S5E). Compared to the control group, the ALI group showed a marked upregulation of M1-related marker genes, while M2-related genes were significantly downregulated (Fig. 9E, Figure S5F). To investigate the importance of Il1r2, we overexpressed Il1r2 in mice from the ALI group and examined inflammatory markers in lung tissue using Western blotting. Lung tissues from ALI mice displayed significant M1 polarization, which was markedly alleviated by Il1r2 overexpression (Fig. 9F-H, Figure S6D, E). Concurrently, overexpression of Il1r2 reduced inflammatory marker levels in lung tissue (Fig. 9I-Q, Figure S6F-N).

## Discussion

ALI/ARDS are severe respiratory conditions characterized by widespread inflammation and impaired gas exchange, typically triggered by factors such as infections, trauma, or systemic diseases^45^. These conditions place a significant burden on the respiratory system and are associated with high mortality rates. While early detection and ventilatory support remain the cornerstone of treatment, a deeper understanding of the cellular and molecular mechanisms underlying these diseases is essential for improving patient outcomes^46^. To explore the potential causes of ALI and gain insights into its pathogenesis, we integrated various models, including lung transplantation, liver transplantation, sepsis, and LPS-induced injury. This approach allowed us to investigate the differential gene expression and immune response patterns, with the ultimate goal of identifying potential diagnostic markers and understanding the role of immune cell infiltration in ALI development.

Through differential gene expression analysis between the ALI and control groups, we identified 197 DEGs, of which 177 were upregulated and 20 downregulated. GSEA revealed that these DEGs were predominantly associated with immune response regulation, inflammation, and cellular stress pathways. Using WGCNA and machine learning algorithms, including SVM-RFE, LASSO, and RF, we identified four key genes—Cebpd, Hspa12b, Pim1, and Il1r2—which were further validated through ROC curve analysis, demonstrating their potential as diagnostic biomarkers for ALI.

Cebpd (CCAAT/enhancer-binding protein delta) plays a crucial role in regulating inflammation and cellular stress, contributing to ALI pathogenesis by modulating pro-inflammatory cytokines such as TNF-α and IL-6. Yan’s studies have shown that Cebpd is essential for the production of these cytokines in LPS-stimulated alveolar macrophages, highlighting its involvement in inflammation^47^. Hspa12b (Heat Shock Protein Family A Member 12B), a molecular chaperone, helps protect cells from stress-induced damage and modulates inflammation, playing a protective role in ALI. Zhang’s results has suggested that managing Hspa12b expression could provide therapeutic benefits, particularly in sepsis-induced ALI^48^. Pim1 (Proto-Oncogene), a serine/threonine kinase, regulates cell survival and proliferation, and its dysregulation may exacerbate inflammation, contributing to ARDS progression. Cao’s findings reveal that inhibiting Pim1 protects against endotoxin-induced ALI by modulating the ELK3/ICAM1 axis on pulmonary microvascular endothelial cells, suggesting it as a potential therapeutic target^49^. Il1r2 acts as a decoy receptor for IL-1β, reducing its inflammatory effects, and plays a key role in modulating the immune response in ALI/ARDS. He et al. discovered that a secondary upregulation of IL-1β-IL-1R signaling drives the pyroptosis of alveolar macrophages and intensifies lung injury in response to LPS^50^. Pyrillou et al. demonstrated the critical role of IL-1R2 in regulating both innate and adaptive immunity through their studies with Il1r2^−/−^ mice^51^. Collectively, these genes contribute to the complex molecular landscape of ALI/ARDS, impacting inflammation, cell stress responses, and disease progression.

Immune cell infiltration analysis, performed using the CIBERSORT algorithm, revealed significant differences in the immune cell populations between the ALI and healthy control groups. Specifically, the ALI group exhibited increased infiltration of monocytes, DCs, and neutrophils, while B cells, T cells, and NK cells were less abundant. This shift in immune cell composition reflects the immune system’s response to lung injury, with monocytes and neutrophils playing key roles in inflammation and tissue damage. Zheng’s team characterized the single-cell landscape in the lung and spleen following SARS-CoV-2 infection and revealed the crucial role of neutrophil-mediated lung immunopathology in SARS-CoV-2-induced severe pneumonia^52^. The decrease in B cells, T cells, and NK cells may be due to immune exhaustion caused by intense inflammation, impairing their function and recruitment. We also observed that Il1r2 was highly enriched in neutrophils, particularly in the N4 subset, which plays a critical role in both the initiation and resolution of inflammation. In the early stages of inflammation, Il1r2 upregulation in the N4 subset helps regulate the immune response by inhibiting excessive IL-1β release, thus preventing immune dysregulation. In later stages, sustained Il1r2 expression may contribute to the resolution of inflammation, preventing chronic inflammation. These findings suggest that Il1r2 acts as a “temporal regulator” of immune responses, modulating both the initiation and resolution of inflammation.

Additionally, we performed hdWGCNA to explore the role of macrophage M6 in exerting anti-inflammatory effects, which are closely linked to neutrophils through Il1r2. Wang et al. demonstrated that mimicking an exogenous “aged” signal selectively triggers macrophage-mediated clearance of activated neutrophils, thereby preventing excessive inflammation and tissue damage in models of acute lung injury and severe acute pancreatitis^53^. Our Cellchat analysis further revealed that N4-macrophages are enriched in the Ptprc-Mrc1 signaling pathway, which is associated with macrophage polarization, particularly the M1-to-M2 transition in the ALI model. This finding is in line with previous research by Ochando’s team, which showed that neutrophil-derived CSF1 regulates macrophage polarization and proliferation, highlighting the crucial cross-talk between neutrophils and macrophages in immune response regulation^54^. In vivo, overexpression of Il1r2 in mice significantly reduced lung inflammation while promoting M2 polarization. Taken together, these results suggest that Il1r2 not only alleviates local inflammation by competing with IL-1β but also regulates neutrophil-macrophage signaling, further balancing the immune response.

While our study offers valuable insights into the pathogenesis of ALI/ARDS, several limitations should be addressed in future research. First, the reliance on publicly available datasets may introduce biases, such as variability in gene expression across different ALI models due to differences in sample handling and processing. Second, the variability observed in immune cell infiltration analysis may reflect factors like cellular heterogeneity or experimental conditions. Further research is needed to validate these findings in clinical settings and to explore the molecular interactions between the identified core genes and immune cells in more depth.

## Conclusion

In summary, we identified Il1r2 as a key regulator in ALI, particularly in neutrophils. Our experimental validation demonstrated that Il1r2 modulates macrophage polarization by reducing pro-inflammatory M1 and promoting anti-inflammatory M2 phenotypes, thereby alleviating lung inflammation. These findings highlight Il1r2’s critical role in controlling immune responses during ALI. Future studies will focus on further elucidating the molecular mechanisms of Il1r2 and exploring its therapeutic potential in inflammatory lung diseases.

## Supplementary Information

Below is the link to the electronic supplementary material.

Supplementary Material 1

## Data Availability

The sequencing data generated in this study (GSE6730, GSE2411, GSE269740, GSE222957, GSE235367, and GSE216943) have been deposited in the GEO database (https://www.ncbi.nlm.nih.gov/geo/query/acc.cgi).
