# Supplementary material for: Neutrophil-derived Il1r2 modulates inflammation and alleviates acute lung injury by promoting M2 macrophage polarization
Source: Sci Rep. 2025 Nov 10;15:39204. doi: 10.1038/s41598-025-14278-4 (PMC12603179; doi:10.1038/s41598-025-14278-4)
Supplement: Supplementary file 1 — Supplementary Material 1 [file 41598_2025_14278_MOESM1_ESM.docx]

**Neutrophil-Derived Il1r2 Modulates Inflammation and Alleviates Acute Lung Injury by Promoting M2 Macrophage Polarization**

Weiwei Ding^1^, Hui Zhang^1^, Bing Li^1^, Xiaodong Xu^1^,Yitian Yang^1^, Luyao Zhang^1*^

^1^Department of Anesthesia and Perioperative Medicine, Henan Provincial People's Hospital, Zhengzhou University, Zhengzhou 450003,China

^*^**Corresponding author:** Luyao Zhang, Department of Anesthesiology and Perioperative Medicine, Henan Provincial People's Hospital, Zhengzhou 450003, China. Email: luyaozhang@126.com

**Supplementary Table 1**: Dataset information

| GEO dataset | Platform | Model | Lung injury | Control |
| --- | --- | --- | --- | --- |
| GSE6730 | GPL339 | RIR | 3 | 3 |
| GSE2411 | GPL339 | LPS | 6 | 6 |
| GSE269740 | GPL24247 | Sepsis | 3 | 3 |
| GSE222957 | GPL23479 | LIR | 3 | 4 |
| GSE216943 | GPL24247 | LPS | 6 | 6 |
| GSE235367 | GPL30215 | HIR | 1 | 1 |

RIR, Renal ischemia reperfusion. LPS, Lipopolysaccharide. LIR, lung ischemia reperfusion. LIR, Hepatic ischemia reperfusion.

**Supplementary Table 2**: Primer sequences for the amplification

| Primer name | Primer sequences（5'-3') |
| --- | --- |
| Cebpd |  |
| 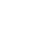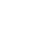Sen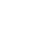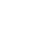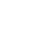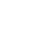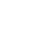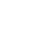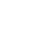se primer | CCGAGCCCTTCTCCATCATT |
| Antisense primer | CCACCATCAGCTGGTCCTTT |
| Hspa12b |  |
| 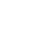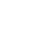Sense primer | CAGGAGTGAGGACGAGGAGG |
| Antisense primer | CAGGAGTGAGGACGAGGAGG |
| Pim1 |  |
| 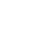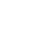Sense primer | TTCCTGCCAGATGACAGCAG |
| Antisense primer | GGACACAGCGAGATGGTGAA |
| Il1r2 |  |
| 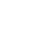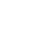Sense primer | AGTCCAGCTCCTGTCGACCT |
| Antisense primer | GCTCTCCGAGTGGGACATTT |
| β-actin |  |
| 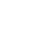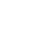Sense primer | GTGACGTTGACATCCGTAAAGA |
| Antisense primer | GTAACAGTCCGCCTAGAAGCAC |

IL, Interleukin

**Supplementary Table 3**: module Trait Cor

|  | Con | ALI |
| --- | --- | --- |
| MEblue | 0.725933154 | -0.725933154 |
| MEturquoise | 0.934468413 | -0.934468413 |
| MEyellow | 0.199956715 | -0.199956715 |
| MEbrown | 0.74475098 | -0.74475098 |
| MEgreen | 0.486081443 | -0.486081443 |
| MElightgreen | -0.164801191 | 0.164801191 |
| MEmagenta | -0.048811482 | 0.048811482 |
| MEtan | -0.099593559 | 0.099593559 |
| MEpurple | 0.25077962 | -0.25077962 |
| MEgrey60 | 0.186411461 | -0.186411461 |
| MEsalmon | 0.56545221 | -0.56545221 |
| MEblack | 0.03011179 | -0.03011179 |
| MElightcyan | -0.427770215 | 0.427770215 |
| MEcyan | -0.670660466 | 0.670660466 |
| MEpink | -0.555214073 | 0.555214073 |
| MEred | -0.491508862 | 0.491508862 |
| MEgreenyellow | -0.641720719 | 0.641720719 |
| MEmidnightblue | -0.354264989 | 0.354264989 |
| MEgrey | 0.058692537 | -0.058692537 |

**Supplementary Table 4**: module Trait *P* value

|  | Con | ALI |
| --- | --- | --- |
| MEblue | 3.80E-06 | 3.80E-06 |
| MEturquoise | 1.54E-14 | 1.54E-14 |
| MEyellow | 0.280810927 | 0.280810927 |
| MEbrown | 1.55E-06 | 1.55E-06 |
| MEgreen | 0.005564116 | 0.005564116 |
| MElightgreen | 0.375646216 | 0.375646216 |
| MEmagenta | 0.794279196 | 0.794279196 |
| MEtan | 0.59399583 | 0.59399583 |
| MEpurple | 0.173588196 | 0.173588196 |
| MEgrey60 | 0.315340079 | 0.315340079 |
| MEsalmon | 0.000916946 | 0.000916946 |
| MEblack | 0.872249432 | 0.872249432 |
| MElightcyan | 0.016372174 | 0.016372174 |
| MEcyan | 3.65E-05 | 3.65E-05 |
| MEpink | 0.00118672 | 0.00118672 |
| MEred | 0.004984123 | 0.004984123 |
| MEgreenyellow | 9.99E-05 | 9.99E-05 |
| MEmidnightblue | 0.050541837 | 0.050541837 |
| MEgrey | 0.753803852 | 0.753803852 |

**Supplementary Figure1**

**
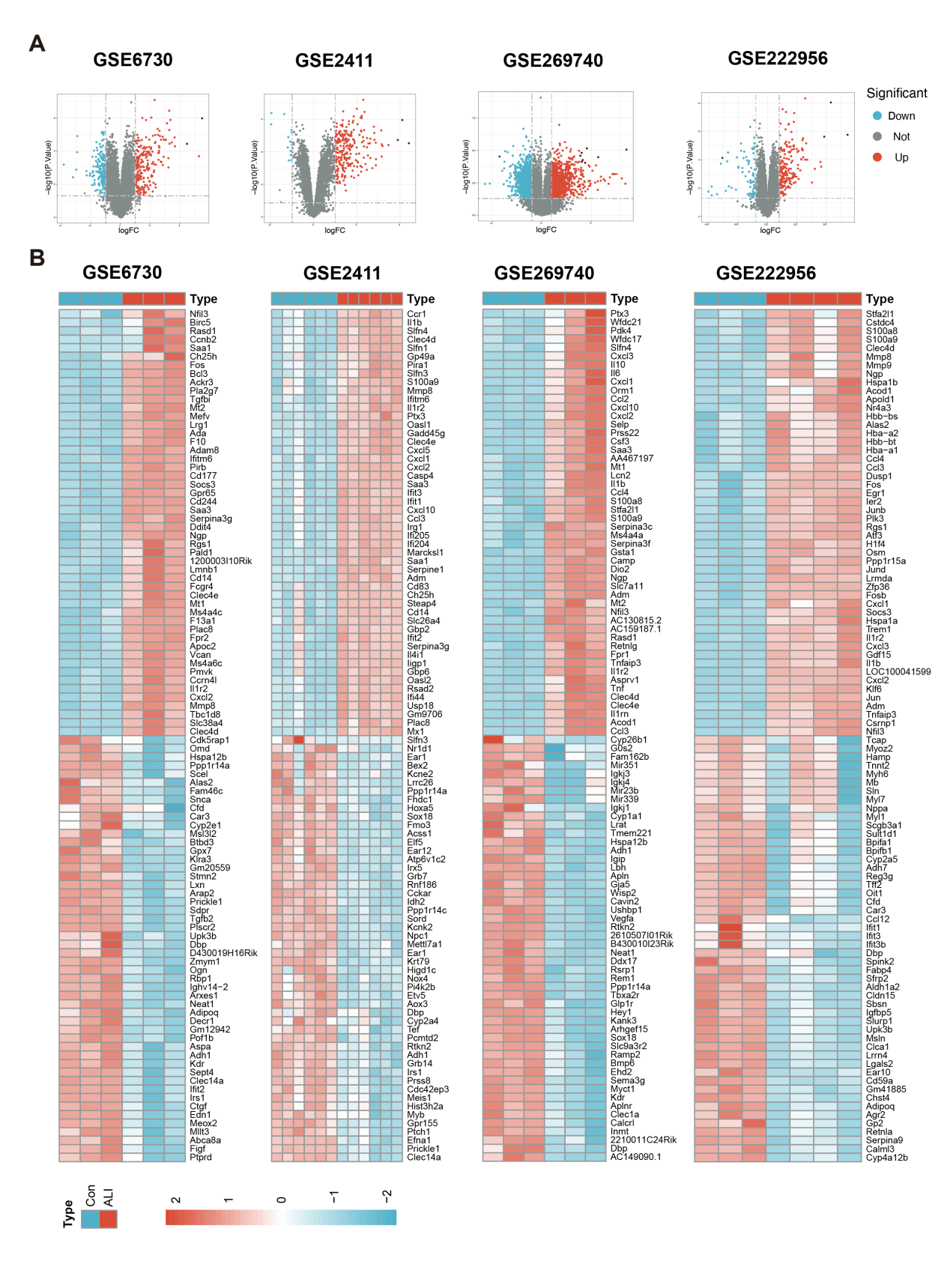
Fig S1.**(A) Volcano plot showing DEGs between ALI and control samples. (B) Heatmap showing the top 50 up- and down-regulated genes. DEGs, Differentially expressed genes.

**Supplementary Figure2**

**
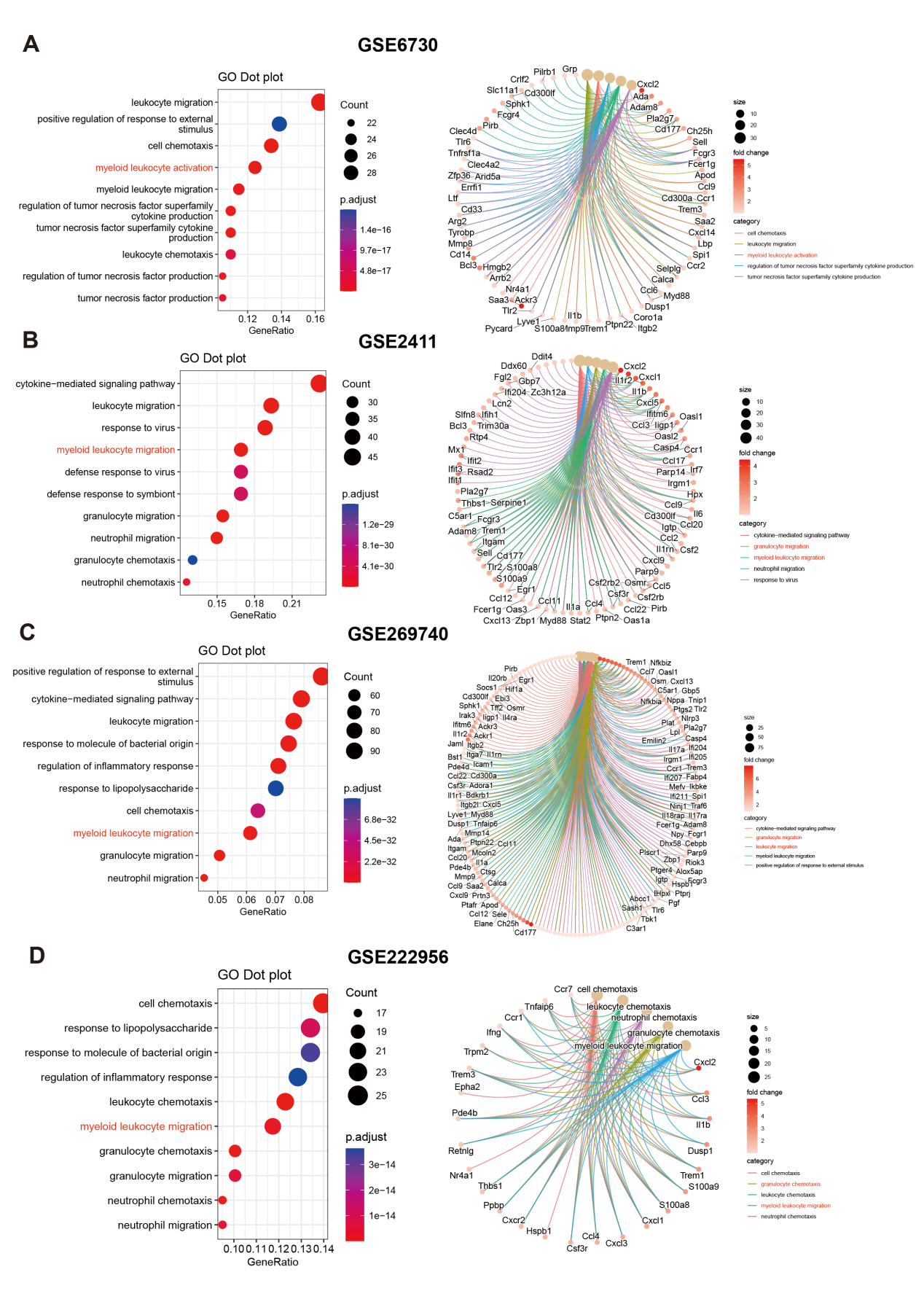
Fig S2.**(A) Top 10 GO enrichment pathways (left) and related genes (right) for the top 5 pathways in GSE6730. (B) Top 10 GO enrichment pathways (left) and related genes (right) for the top 5 pathways in GSE2411. (C) Top 10 GO enrichment pathways (left) and related genes (right) for the top 5 pathways in GSE269740. (D) Top 10 GO enrichment pathways (left) and related genes (right) for the top 5 pathways in GSE222956. GO, Gene ontology.

**Supplementary Figure3**

**
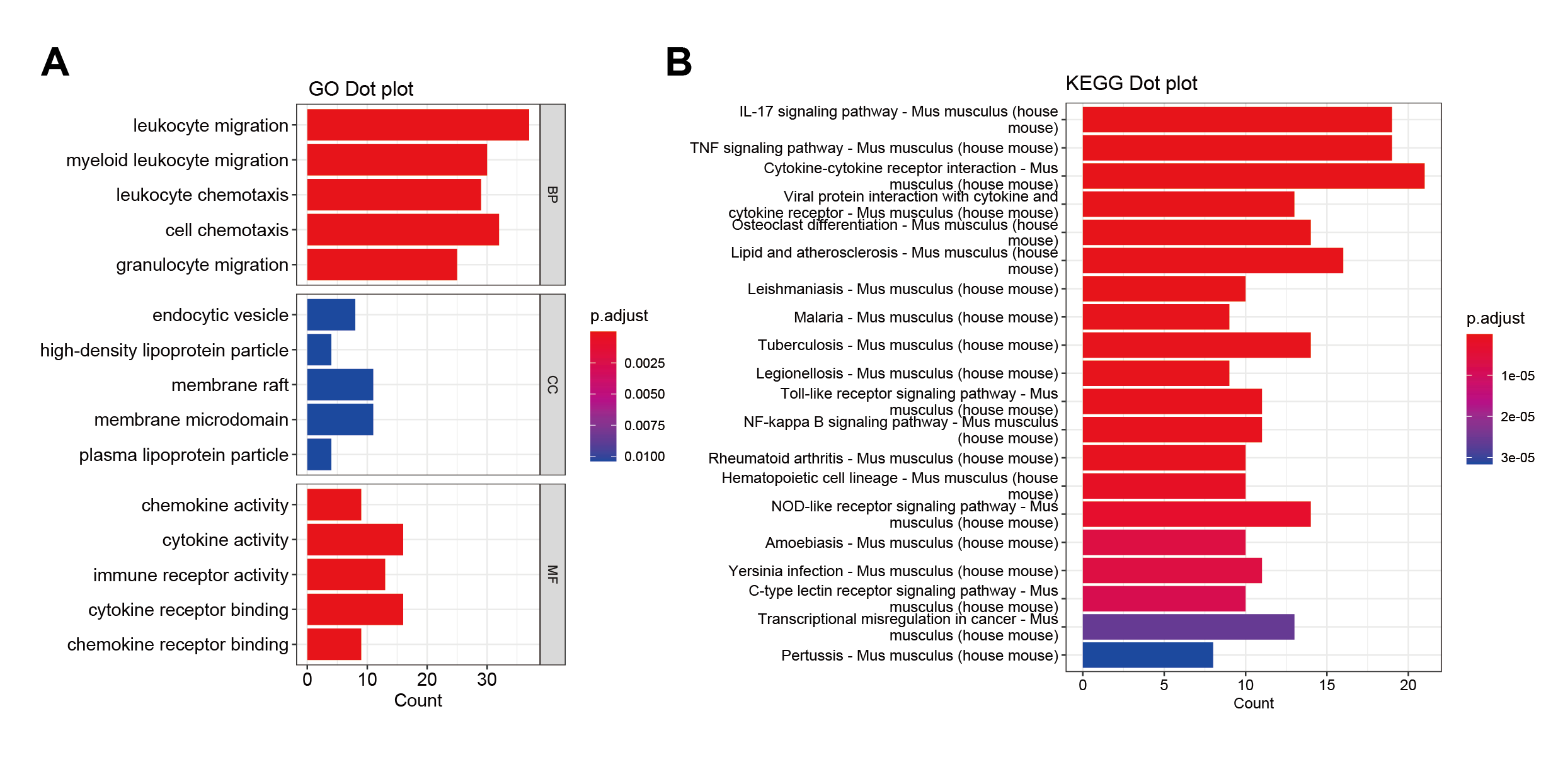
Fig S3.**(A) Top 5 GO (BP, CC, MF) enrichment pathways. (B) Top 20 KEGG enrichment pathways. GO , Gene Ontology. KEGG, Kyoto Encyclopedia of Genes and Genomes. BP, Biological process. CC, Cellular component. MF, Molecular function.

**Supplementary Figure4**

**
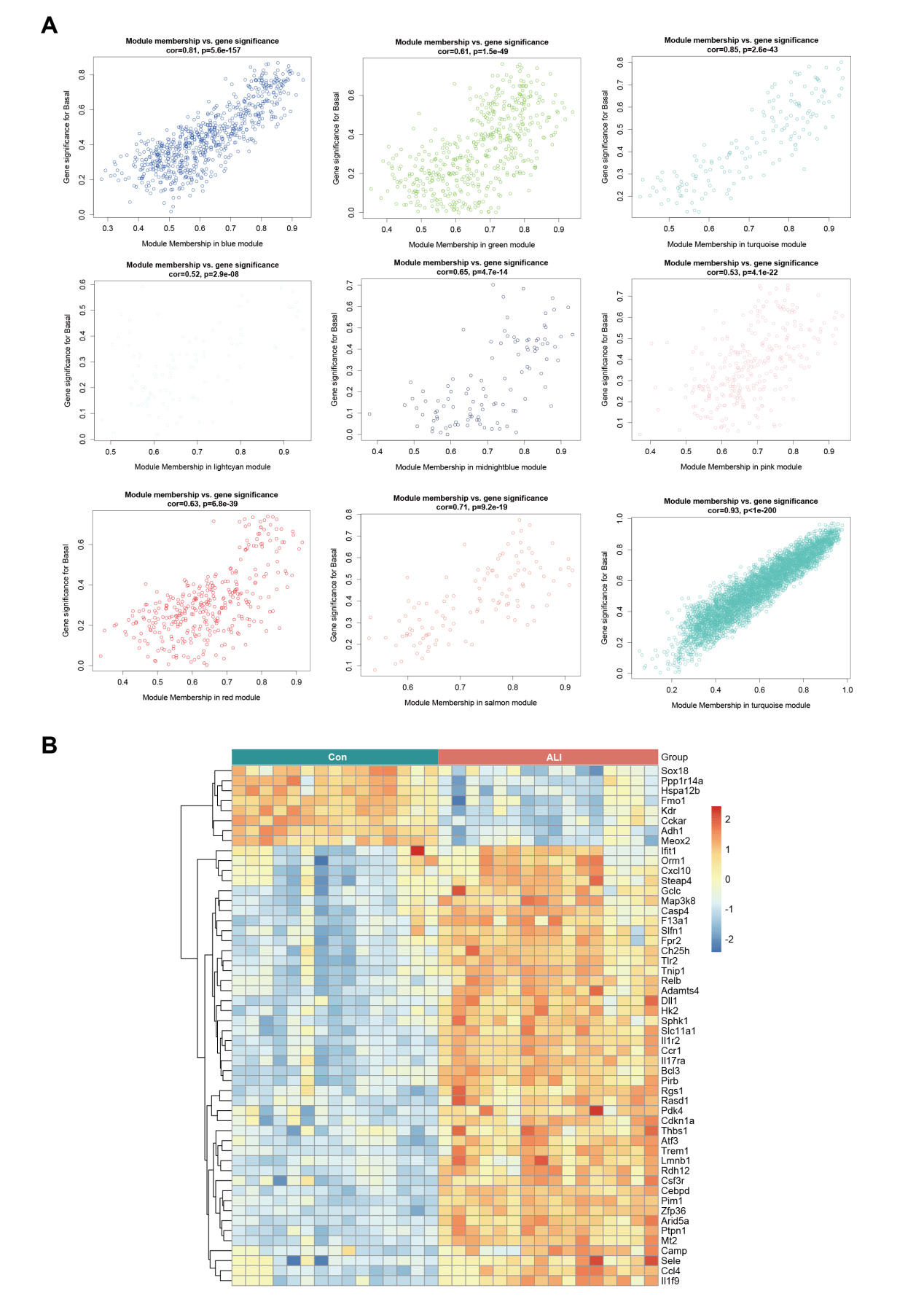
**

**Fig S4.**(A) The significance of genes related to ALI in module (a dot represents the genes in the module). (B) Heatmap showing the expression levels of 52 differentially expressed genes in the ALI and Con groups. ALI, Acute lung injury. Con, Control.

**Supplementary Figure5**

**
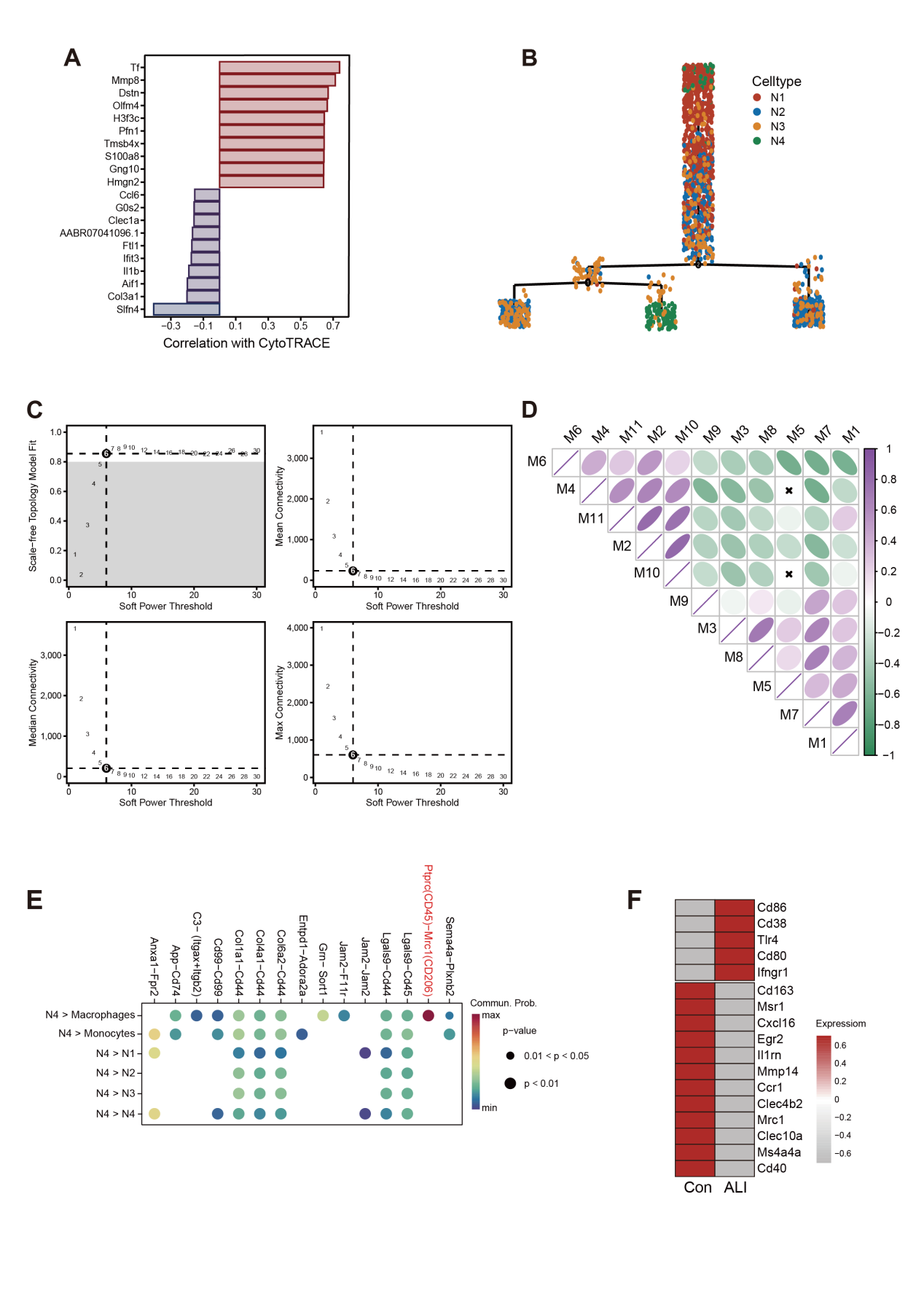
**

**Fig S5.**(A) Visualization of the top 10 genes most positively and negatively correlated with CytoTRACE scores. Genes are ranked based on their correlation coefficients. Positively correlated genes are associated with lower differentiation states (higher developmental potential), whereas negatively correlated genes are linked to more differentiated cell states. (B) The scatter plot shows the differentiation trajectory of the neutrophil subgroup. (C) Weighed gene co-expression network analysis was constructed among macrophages. (D) Correlation analysis between modules. (E) Receptor design for N4 neutrophils and other cell types as signal senders. (F) Heatmap showing the expression levels of macrophage polarization-related genes in ALI and control samples. All data are expressed as mean ± SD, *P* < 0.05 were considered statistically significant differences.

**Supplementary Figure6**

**
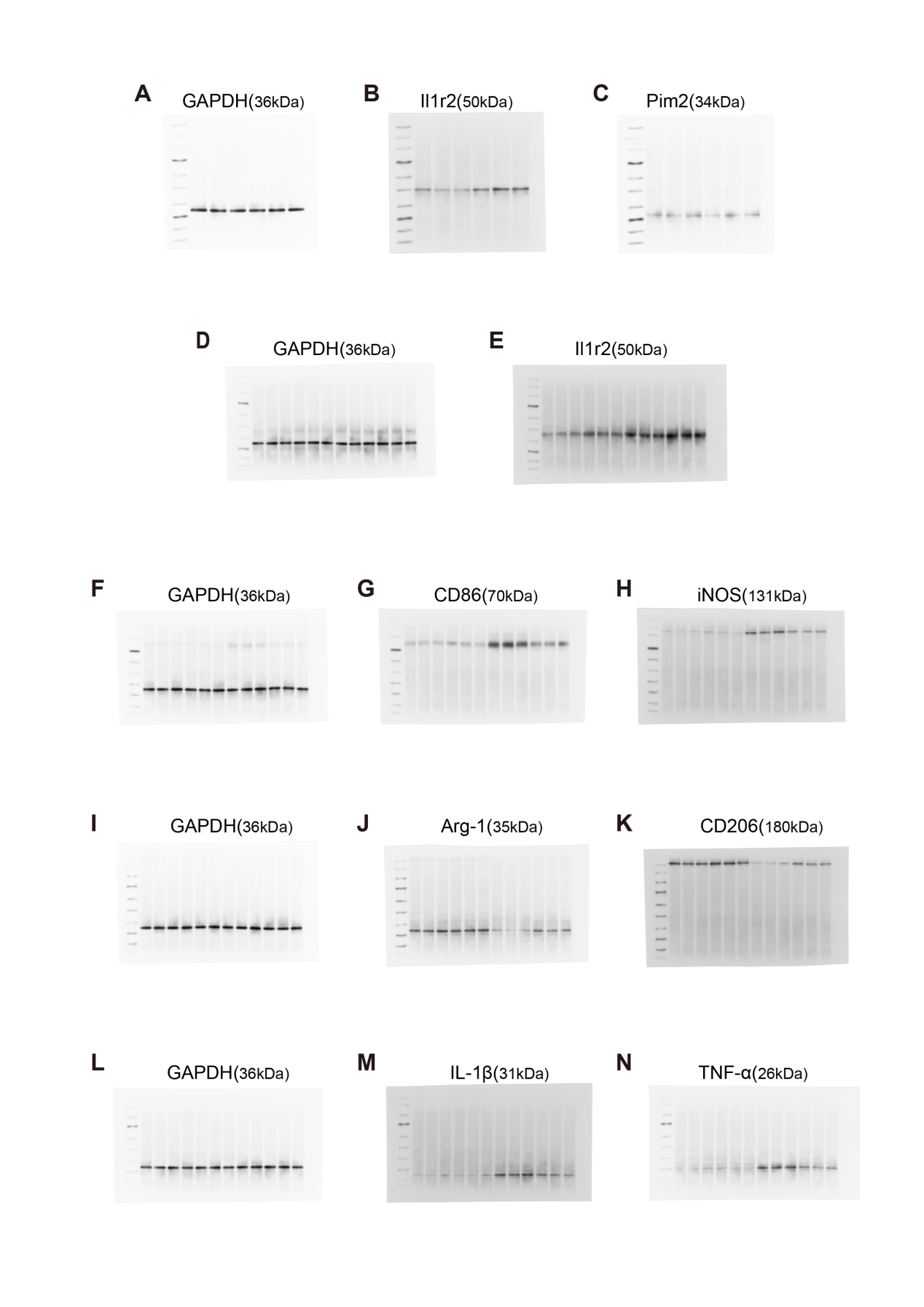
Fig S6.** (A-N) Full-length blots.
